# Network analysis predicts pembrolizumab response in advanced NSCLC with PD-L1 < 50%

**DOI:** 10.1186/s12935-026-04268-5

**Published:** 2026-03-26

**Authors:** Mario Occhipinti, Antonella Cruoglio, Silvia Marchesi, Arsela Prelaj, Marco Petrilli, Aurelia Rughetti, Paolo Ambrosini, Elisabetta Ferretti, Claudia Proto, Monica Ganzinelli, Marta Brambilla, Laura Mazzeo, Teresa Beninato, Filippo de Braud, Marina Chiara Garassino, Lorenzo Farina, Giuseppe Lo Russo, Manuela Petti

**Affiliations:** 1 Medical Oncology Department, Fondazione IRCCS Istituto Nazionale Dei Tumori, Milan, Italy; 2 MSc Degree Program In Data Science, Sapienza University of Rome, Rome, Italy; 3 Institute of Endotypes in Oncology, Metabolism, and Immunology, National Research Council (CNR), Naples, Italy; 4 MSc Degree Program In Statistical Science, Sapienza University of Rome, Rome, Italy; 5 Department of Experimental Medicine, Sapienza University of Rome, Rome, Italy; 6 Thoracic Oncology Program, Department of Medicine, Section of Hematology/Oncology, The University of Chicago, Chicago, USA; 7 Department of Computer, Control, and Management Engineering, Sapienza University of Rome, Rome, Italy

**Keywords:** Data integration, Differential co-expression network, Network analysis, NSCLC, PD-L1, Biomarkers

## Abstract

**Background:**

The efficacy of single-agent immune checkpoint inhibitors as a first-line treatment for advanced non-small cell lung cancer (aNSCLC) patients with PD-L1 Tumor Proportion Score (TPS) < 50% remains variable. Network analysis is promising in addressing tumor biology and behavior, potentially predicting therapeutic response.

**Methods:**

This study, based on the PEOPLE trial (NCT03447678) data, explores network analysis for predictive biomarker discovery in immunotherapy response. Utilizing circulating immune profiling (CIP) and gene expression profiling (GEP), key immune cells and gene interactions were identified.

**Results:**

Our findings confirm the central role of natural killer (NK) cells, with elevated baseline levels associated with a favorable response. Differential co-expression network (DCN) analysis of GEP identified 23 hub genes, with enrichment analysis linking CD48 to immune-related processes. Patient similarity network (PSN) analysis identified two patient clusters with significantly different survival outcomes. The integrated model outperformed single-layer approaches, supporting the added value of combining GEP and CIP data.

**Conclusions:**

Despite limitations such as a non-randomized design and small sample size, the study’s innovative network approach provides valuable insights. The results suggest that baseline NK cell subsets and specific gene evaluations could guide personalized treatment strategies, optimizing the use of pembrolizumab in aNSCLC patients with PD-L1 TPS < 50%.

**Supplementary Information:**

The online version contains supplementary material available at 10.1186/s12935-026-04268-5.

## Background

In the ever-evolving landscape of cancer treatment, immunotherapy stands as a cornerstone for metastatic and locally advanced non-small cell lung cancer (NSCLC) lacking driver alterations. Despite its limitations, programmed death-ligand 1 (PD-L1) Tumor Proportion Score (TPS) remains the primary predictor of activity [1–3]. Clinical practice currently selects patients for immunotherapy based on the absence of driver molecular alterations and a PD-L1 TPS ≥ 50%, guiding the use of first-line single-agent immunotherapy or immunotherapy-chemotherapy combinations. However, refining patient selection strategies remains a significant challenge. The Phase II PEOPLE trial (NCT03447678) aimed to address this by assessing new biomarkers in advanced NSCLC (aNSCLC) patients with PD-L1 TPS < 50% treated with first-line pembrolizumab monotherapy [4]. Recent advances in high-throughput technologies have generated vast "omics" datasets, revolutionizing our understanding of biological complexity. To manage the dimension and complexity of biological data, innovative tools are required, and network formalism has emerged as a powerful paradigm. Network medicine has emerged as a systems-level framework based on interdisciplinary collaboration to model complex biological interactions in cancer. This holistic approach to understanding complex diseases emphasizes the intricate web of interactions within biological systems [5]. By mapping the relationships between genes, proteins, cells, and even patients, network medicine enhances our understanding of diseases like cancer and paves the way for personalized and targeted therapeutic strategies [6]. The advent of immunotherapy, particularly immune checkpoint inhibitors (ICIs) such as pembrolizumab, has ushered in a new era of cancer treatment [7]. However, the clinical efficacy of these therapies remains variable, necessitating a deeper understanding of the molecular and immune landscape of individual patients. This is where network medicine becomes invaluable. Using computational techniques, researchers can construct intricate networks that reflect the complexity of biological systems. These networks serve not only as maps of cellular and molecular interactions but also as tools for identifying key players or biomarkers that influence disease progression and treatment response. In cancer immunotherapy, network medicine provides a unique way of exploring the intricate interplay between the immune system, tumor cells, and the microenvironment [8, 9].

This study delves into the world of aNSCLC patients with PD-L1 TPS < 50% by analyzing circulating immune profiling (CIP) and gene expression profiling (GEP) data from the Phase II PEOPLE clinical trial [4] with network-based approaches. This analysis aims to uncover novel biomarkers or pathways critical for response to immunotherapy. These biomarkers could serve as predictive indicators to help clinicians tailor treatments to individual patients, maximizing efficacy while minimizing side effects.

The objectives of this work are twofold: firstly, to characterize responder and non-responder patients by analyzing the detailed interaction maps constructed from the CIP and GEP data, and secondly, to identify potential predictive biomarkers within these networks. To achieve these goals, we investigated the circulating and tissue levels both separately and by integrating them through a multi-layer network model.

## Methods

### Study population, procedures and objectives

PEOPLE (NCT03447678) is a prospective, monocentric, open-label phase II trial conducted at Fondazione IRCCS Istituto Nazionale dei Tumori (INT) in Milan. It evaluated pembrolizumab at a flat dose of 200 mg administered intravenously every three weeks in treatment-naïve aNSCLC patients with PD-L1 TPS < 50%. Full inclusion and exclusion criteria and treatment procedures have already been described elsewhere [4, 10]. Baseline tumor tissue was used for GEP. CIP analyses were performed on blood samples collected at baseline and at radiological evaluation after two cycles of treatment. The local Ethical Committee approved the trial protocol and all related amendments. The trial was conducted in accordance with the International Conference for Harmonization Guidelines on Good Clinical Practice and the Declaration of Helsinki. All patients provided their written informed consent before enrollment.

Details of patients’ characteristics and survival analysis are provided in Supplementary Part 1. Supplementary Part 2 describes the methodology for CIP and GEP analysis and data preprocessing.

### CIP correlation network analysis

Correlation networks were derived from CIP data using the Spearman correlation measure, for four distinct groups: (1) responders at baseline, (2) non-responders at baseline, (3) responders after treatment and (4) non-responders after treatment. Correlation coefficients with absolute values exceeding 0.55 and FDR-adjusted p-values less than 0.05 were retained: in the resulting networks, edge weights represent significant correlation coefficients and no-links denote non-significant correlations. These weighted networks capture relationships among immune cell populations.

### Differential expression analysis

Differential expression analysis (DEA) is defined as a comparison between experimental groups to identify genes with significant expression changes between conditions. In this study, the DEA was conducted on the raw counts of 438 genes obtained from 44 patients with GEP available. The aim was to identify genes that exhibit significant expression differences across the two response groups. The analysis of differential expression genes (DEGs) was conducted using DESeq2 library (v 1.40.2) [11]. Genes with a log2-fold change > 1 and an FDR ≤ 0.05 were considered differentially expressed (Supplementary Part 1).

### Co-expression network and differential co-expression network analysis

Two distinct co-expression networks, one for responders and another for non-responders, were constructed using gene expression data available exclusively at baseline. Spearman correlation coefficient for all possible pairs among the 438 genes was calculated as a measure of co-expression: Spearman coefficients exceeding the absolute value of 0.7 were retained and FDR-adjusted p-values < 0.05 ensured the inclusion of significant co-expression relationships in the networks (Supplementary Part 1). Subsequently, differential correlation between the 2 groups was tested based on Fisher’s z-test. We implemented R package DiffCorr [12]: Spearman correlation coefficients were transformed using Fisher Z transformation and z-scores were computed as in Eq. 1.

1 $$Z=\frac{{z}_{A}-{z}_{B}}{\sqrt{\frac{1}{{n}_{A}-3}+\frac{1}{{n}_{B}-3}}}$$

where n_A_ and n_B_ represented sample size for each gene pair under each condition (respectively responders and non-responders). To focus on the most significant changes, only links with absolute z-scores greater than 2 were retained.

### Differential co-expression network topological analysis

Differential co-expression network (DCN) analysis was performed to highlight key genes involved in the most relevant differences between responders and non-responders. The first investigation focused on the identification of genes involved in the highest number of changes in the co-expression pattern. For this purpose we calculated the degree index: hubs of the DCN were identified by selecting the right tail of the degree distribution based on 95th percentile threshold. In addition, we performed the detection of mesoscale network structures applying the Louvain algorithm [13] to our DCN. The objective was to uncover higher-order patterns within the network, moving beyond local connections and focusing on a global perspective.

### DCN functional analysis

To interpret the functional significance of identified network components (hubs and communities), we performed enrichment analysis. This included Gene Ontology (GO) terms and Kyoto Encyclopedia of Genes and Genomes (KEGG) pathways [14–16], aiming to elucidate their biological roles. We employed rigorous methods to mitigate biases, ensuring the robustness and relevance of our findings. Enrichment analysis identifies GO terms and KEGG pathways overrepresented in a gene set based on the hypergeometric test. Given that our analysis was based on an ad-hoc selected panel of genes, rather than the entire transcriptome, we defined a background set as the set of all 438 genes in the DCN. BH correction was applied to account for multiple testing. This enrichment analysis was performed firstly considering the gene set composed of each hub node with its first neighbors, and then considering each detected community.

### Patients similarity network and survival analysis

In a patient similarity network (PSN), the nodes represent individual patients, and edges between nodes indicate the degree of similarity between those patients. The similarity can be defined considering various factors such as clinical features, genomic data, treatment response, or other relevant information. In this study, for the integration of CIP and GEP data, we used Similarity Network Fusion (SNF) [17]. SNF consists of two main steps: constructing a PSN for each data type and integrating these networks into a single similarity network using a nonlinear combination method [6]. SNF is computationally efficient and robust and has been widely and successfully applied to detect molecular subtypes of tumors [18–21]. In the present work, we used the SNFtool R package (v 2.3.1) [17] to first build the two distinct similarity networks (one using gene expression data and another using circulating immune profiling) and then to integrate the two levels of information. In the case of GEP PSN, we defined the patient similarity using the expression profile of hub genes (high-degree nodes of the differential co-expression network), whereas the full set of 31 variables was used for the CIP PSN. The individual layers were then merged in a single network of 38 common patients (20 responders and 18 non-responders) [17]. Spectral clustering on the fused patient network was used to identify subtypes and we selected 2 as the optimal number of clusters, which maximizes the eigengap. Finally, Kaplan–Meier survival curves were generated, allowing for the visualization of differences in OS between patient subgroups. Log-rank tests were employed to determine the statistical significance of observed differences. Moreover, the patient cluster composition was characterized in terms of immunotherapy response (hypergeometric test).

To evaluate the impact of hubs identification, we also performed a sensitivity analysis testing different thresholds on the degree distribution of the DCN. In detail, we considered 75th, 90th, 95th, 98th, and 99th percentile, and, for each threshold, we evaluated the stability of the resulting SNF patient clusters and the mean accuracy (Supplementary Part 1). The 95th percentile has been shown to maximize the trade-off between predictive accuracy and feature reduction, resulting in a set of 23 genes.

Lastly, for validation purposes and to test the importance of data integration (periphery and tissue levels), we also analyzed the networks built using a single data type. In the case of GEP, PSN was also constructed exploiting the whole gene expression profile (438 genes instead of 23). The two networks were again investigated for disease subtyping (spectral clustering) and network-based survival risk prediction (Kaplan–Meier estimate and log-rank test).

### Internal validation

Due to the limited sample size, we performed a Leave One Out Cross Validation (LOOCV) to assess the robustness of the identified network. In this procedure, one patient was iteratively removed from the dataset and the entire process of network construction and clustering process was performed on the remaining N-1 patients. Both the classification accuracy (concordance with clinical response) and the prognostic performance (survival stratification) were evaluated across iterations. To demonstrate the independent prognostic value of the network-derived clusters, we performed a multivariate Cox model, adjusting for standard baseline clinical covariates: Age, Smoking History (Never vs Former vs Current), Histology (Squamous vs Non-Squamous), PD-L1 TPS status (categorized as < 1% vs 1–49%) and ECOG PS (0–1 vs 2). Comparisons of baseline characteristics between clusters were assessed using Fisher’s exact test or Wilcoxon rank-sum test as appropriate.

## Results

### Circulating immune profiling correlation network in responder and non-responder patients

**Fig. 1:**
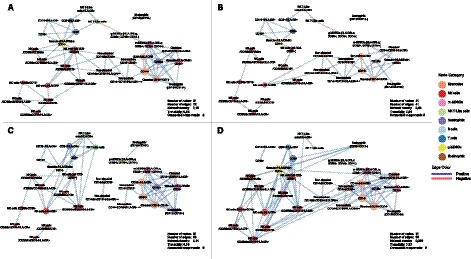
CIP correlation networks with summary statistics. Networks display only significant correlations (Spearman’s |ρ|> 0.55, p < 0.05, FDR-adjusted). **A**: Pre-therapy Correlation Network of responders. **B**: Post-therapy Correlation Network of responders. **C**: Pre-therapy Correlation Network of non-responders. **D**: Post-therapy Correlation Network of non-responders. Node size reflects degree of connectivity; node color denotes immune subset type; edge color represents correlation sign

Pre-therapy and post-therapy CIP correlation networks of responder and non-responder patients are represented in Fig. 1A-D. The pre-therapy responder network (Fig. 1A) consists of 31 nodes (*i.e.*, the 31 immune cell subsets) and 72 edges, 71 of which are positive, with only one negative edge between g-MDSCs and natural killer (NK)-T-like cells. A highly interconnected group of nodes, composed of monocytes, neutrophils, and myeloid suppressor cells, can be observed. Outside of this component, nodes with the highest number of connections are NK cells (all CD3- CD56 +) and Granulo-/HLA-DRdim CD14-. Conversely, the pre-therapy non-responder network (Fig. 1C) has 63 links, all positive. Similar to the pre-therapy responders network, there is a cluster of monocytes, neutrophils, and m-MDSCs. However, in the pre-therapy non-responder network, there is no "bridge" between this cluster and other parts of the graph, leading to distinct separated components. This difference is mainly due to the lack of connections between nodes belonging to the NK cells category and those of other types (e.g., monocytes and g-MDSCs cells).

The post-therapy responder network (Fig. 1B) has a lower density (41 edges). The negative edge between g-MDSCs and NK-T-like cells, found in the pre-therapy responder network, is also present in the post-therapy responder network. However, there are fewer connections between nodes; monocytes and m-MDSCs are connected, whereas neutrophils are only connected among themselves. The post-therapy non-responder (Fig. 1D) network has 86 positive edges, with nodes such as Granulo-/HLA-DRdim CD14-, Classical CD14 + CD16-/HLA-DR + , CD14 + , and CD14 + /HLA-DR + showing the highest number of connections.

Comparing pre-treatment and post-treatment networks of responders, fewer connections between markers are observed post-therapy. The centrality and importance of nodes like Granulo/HLA-DRdim CD14-, NK cells (all CD3- CD56 +), and NK cells/CD56dimCD16 + are diminished (Fig. 1). For non-responders, the pre-therapy network shows disconnected components, whereas the post-therapy network exhibits many connections.

### DCN of gene expression profiling

To molecularly characterize and distinguish responders and non-responders at the tissue level, we analyzed gene expression profiles comparing the two patients’ groups in terms of co-expression patterns. Once the two co-expression networks were obtained, we focused on the construction and analysis of the DCN (responders vs non-responders): in this network, the presence of a link informs about the significant changes in the co-expression between two genes.

### DCN topological analysis

The resulting DCN comprised 1,757 edges (1,020 positive and 737 negative). Positive edges indicate gene pairs exhibiting stronger co-expression in responders compared to non-responders, whereas negative edges reflect gene pairs with reduced co-expression in responders relative to non-responders.

**Fig. 2:**
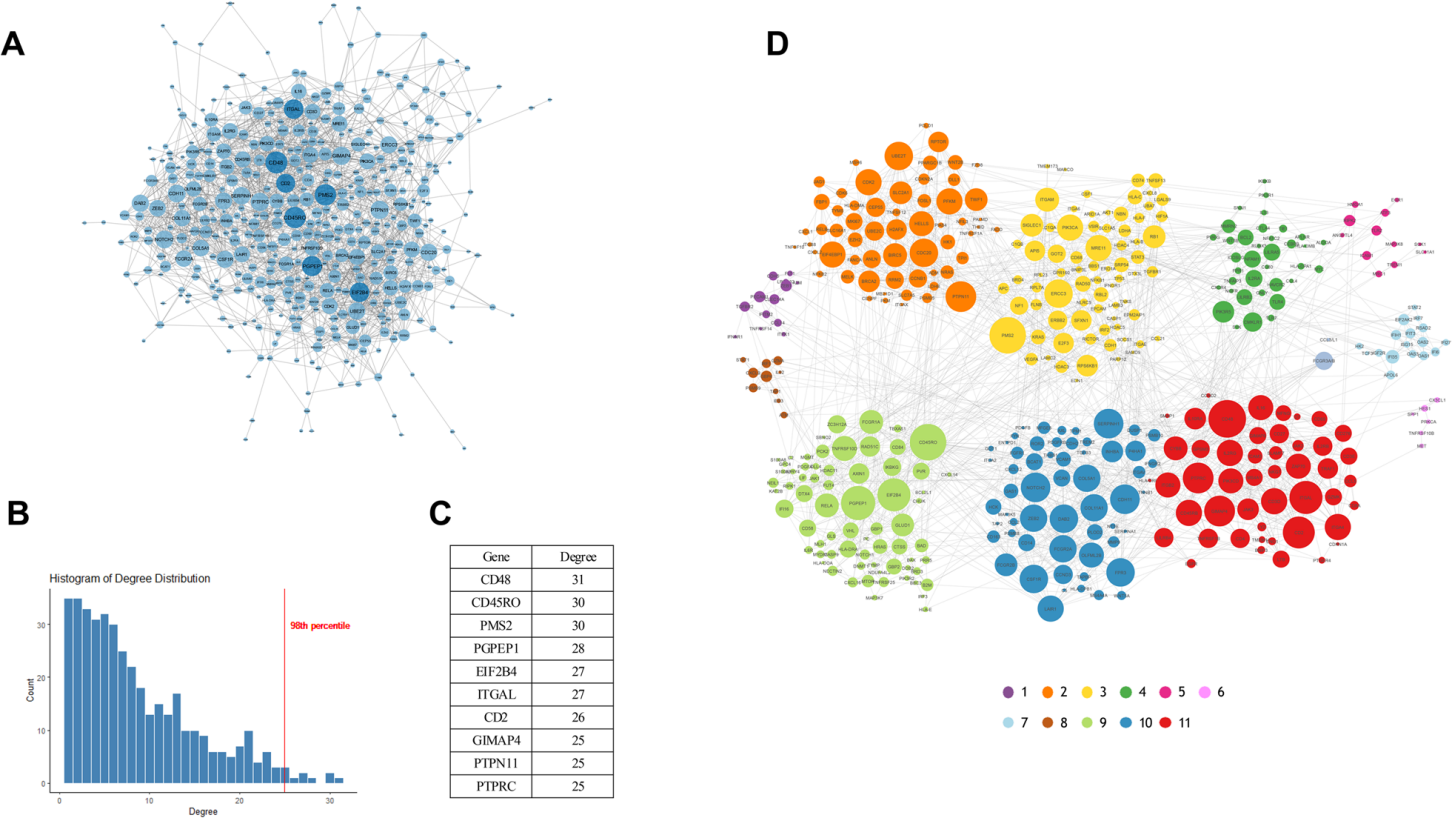
**A**: Differential Co-expression Network. Hubs are highlighted in dark blue. **B**: Node Degree Distribution of the DCN. **C**: List of hub genes in the Differential Co-expression Network and their degree. **D**: Differential Co-expression Network with highlighted community structure. Node color represents community membership. Node size is proportional to node degree

By considering all nodes with a degree greater than the 95th percentile, 23 hub genes were identified (Fig. 2A-C). None of these hubs are among the list of genes individuated by the DEA (Supplementary Part 1).

Applying the Louvain community detection algorithm to our DCN, we identified 11 modules (modularity$Q=0.5$). Module 11 and Module 10 contain the majority of high degree genes within the DCN (Fig. 2D) (Supplementary Part 1). Genes in Modules 10 and 11 are reported in Supplementary Part 1.

### Enrichment analysis

A stringent approach was used to select background genes for enrichment analysis, restricting to the 438 genes resulting from the preprocessing phase. By restricting the background to genes available in this study, we prevented any skewed interpretation of the enrichment results and in drawing reliable conclusions about the functional implications of gene sets in the studied biological processes, independent of any systemic biases.

**Fig. 3:**
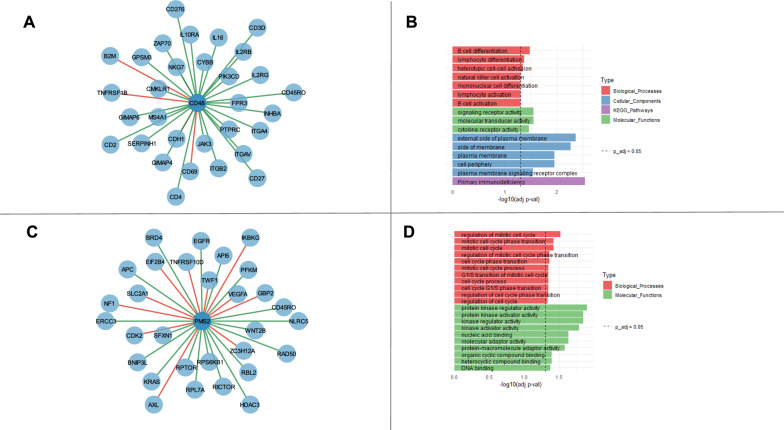
**A**: Subgraph of CD48 and its neighbors in the DCN. Positive connections in green, negative connections in red. **B**: Enrichment Analysis of CD48 positive connections in the DCN. **C**: Subgraph of PMS2 and its neighbors in the DCN. Positive connections in green, negative connections in red. **D**: Enrichment Analysis of PMS2 positive connections in the DCN

No immune response-related enrichment terms of DEGs were found. Similarly, the entire set of hubs was not enriched in any term. However, focusing on the links of each hub, the positive connections (stronger co-expression in responders with respect to non-responder group) of CD48, the hub with the most connections, demonstrated significant associations with immune system functionality (Fig. 3A-B). Conversely, positive connections of PMS2 displayed enrichments primarily associated with cell cycle regulation and molecular activities, proving that not all the hubs are enriched in terms related to immune response (Fig. 3C-D).

**Fig. 4:**
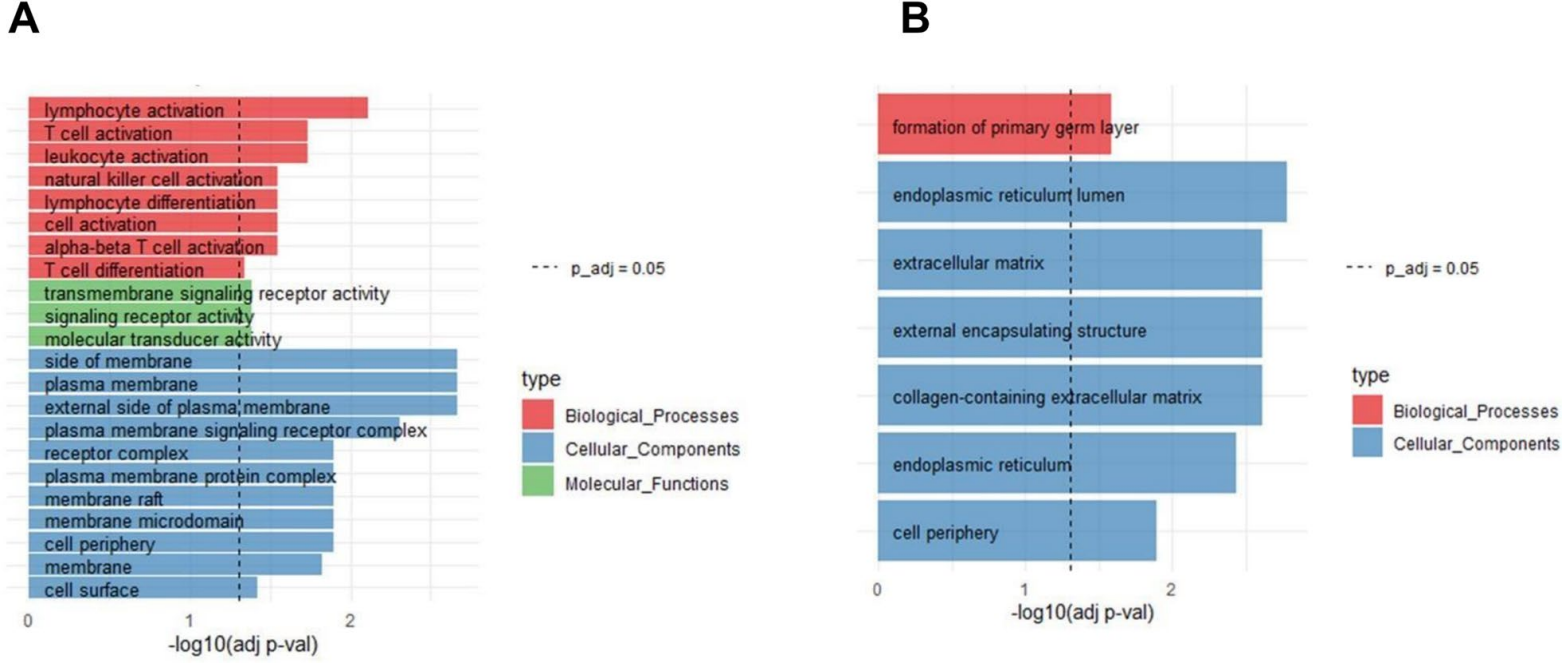
**A**: Enrichment Analysis of Module 11. **B**: Enrichment Analysis of Module 10

In Module 11, CD48, CD2, GIMAP4 and PTPRC are also hubs of the DCN. The enrichment analysis of this set showed terms mainly related to immune responses (Fig. 4A). Conversely, Module 10’s enrichment analysis did not show any relation to immune responses (Fig. 4B).

### Stratification of patient survival based on CIP and GEP integration

**Fig. 5:**
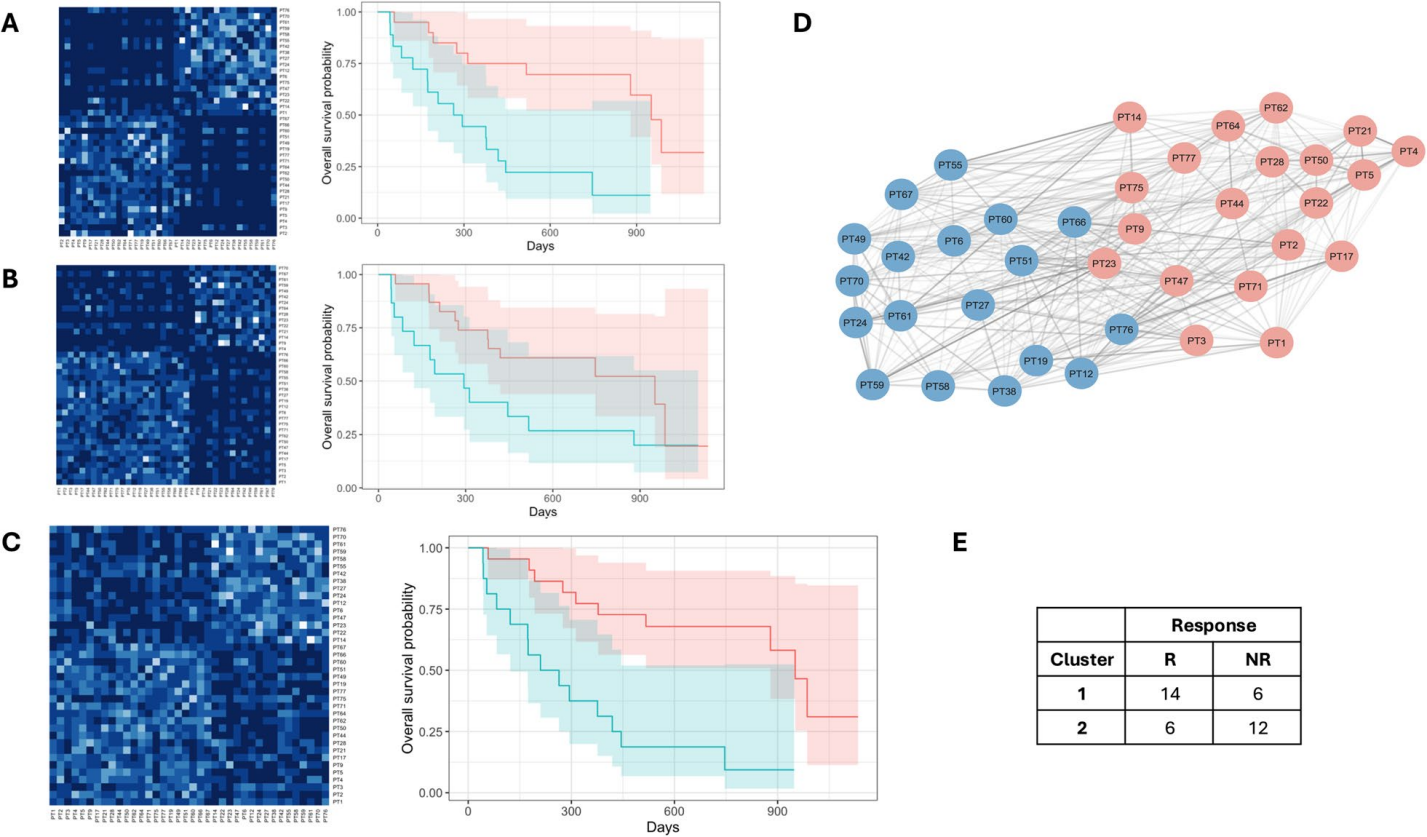
Patient Similarity Network with Similarity Network Fusion. **A**: Left: Gene expression-based PSN for 38 patients. Right: Kaplan–Meier survival analysis for Cluster 1 (red) and Cluster 2 (cyan). Log-rank test p-value = 0.001. **B**: Left: Circulating immune profile-based PSN for 38 patients. Right: Kaplan–Meier survival curves for Cluster 1 (red) and Cluster 2 (cyan). Log-rank test p-value = 0.06. **C**: Left: Integrated PSN using Similarity Network Fusion (SNF). Right: Kaplan–Meier survival analysis for the two clusters. Log-rank test p-value = 3 × 10⁻^4^. Cox regression model: Hazard Ratio (HR) = 4.49, 95% CI [1.85, 10.90], p-value < 0.001. **D**: Patient Similarity Network colored by SNF-derived clusters (Cluster 1: red, Cluster 2: cyan). **E**: Confusion matrix comparing SNF clusters with true response labels (NR = Non-Responders, R = Responders)

To uncover subgroups of patients with shared characteristics and potential similar responses to pembrolizumab, we constructed a multi-layer patient similarity network. Initially, we obtained two separate networks: one based on GEP data focusing on the top 23 hub genes identified in the DCN, and another based on CIP data utilizing all 31 DCN nodes (Fig. 5A-B). Then, we integrated the two layers obtaining the fused similarity network with SNF method (Fig. 5C-D). The resulting clusters provided a comprehensive view of the studied population: Cluster 1 was enriched in responders (hypergeometric test, p-value = 0.0004; 16 responder patients and 6 non-responder patients), while Cluster 2 included 4 responder patients and 12 non-responder patients resulting enriched in non-responders (hypergeometric test, p-value = 0.003). The accuracy rate of this fused network reached 0.74 (Fig. 5E), further highlighting the effectiveness of the identified clusters in capturing underlying similarities. To further evaluate the differences between clusters, we performed Mann–Whitney U tests comparing all the variables in individual GEP and CIP layers. This revealed significant differences in 12 out of 23 genes for gene expression data and 10 out of 31 significant differences among circulating immune data variables (see Supplementary Part 1). Moreover, we employed similarity metrics to validate cluster separation. LOOCV was used to assess the robustness of our findings. The mean classification accuracy (concordance with clinical response) was 0.711 (± 0.028) (Supplementary Part 1). In addition, Kaplan–Meier survival curves showed a statistically significant separation (p < 0.05) in all LOOCV iterations.

Of particular interest, the survival outcomes in the identified clusters showed remarkable disparities, with Cluster 1 exhibiting a median survival of 32 months and Cluster 2 showing a median survival of 8 months (Fig. 5C). A Log-rank test revealed a highly significant p-value of 3 × 10^–4^ and with hazard ratio (HR) = 4.49, emphasizing the prognostic relevance of these distinct clusters in the context of patient survival. This statistical significance underscores the potential of these clusters as prognostic markers, providing valuable insights into the response dynamics to pembrolizumab. The analysis of both single-layer networks (CIP PSN and GEP PSN separately) also revealed a division of patients into two distinct clusters. However, the comparison of these clusters composition with actual responders and non-responders groups showed lower accuracy rate (0.68 for the GEP network and 0.50 for the CIP network) than that reached with the integration of the two levels (fused PSN). The survival outcomes in the identified clusters exhibited significant disparities only for GEP network: HR of 4.20 and Log-Rank test p-value = 0.002 for the GEP PSN, and HR = 2.13and Log-Rank test p-value = 0.06 for the CIP PSN (Supplementary Part 1).

To confirm that this stratification was not influenced by potential confounding factors, we compared the baseline clinical traits of the two clusters. Supplementary Part 1 shows no significant statistical difference in Age, Sex, Smoking Status, Histology or PD-L1 Status. Notably, the proportion of PD-L1 negative (< 1%) and low-positive (1–49%) patients was comparable across the two groups (71% vs 68% and 29% vs 32% respectively), indicating that the network identifies a biological signal independent of PD-L1 expression levels.

We further validated the prognostic independence of the network signature through multivariate analysis. Even after adjusting for Age, Smoking History, Histology, PD-L1 Status and ECOG Performance Status, the clustering derived from the network remained a statistically significant predictor of survival (HR = 3.83, 95% CI [1.32 – 11.2], p = 0.015). ECOG PS emerged as the only other significant prognostic factor in this cohort (HR = 5.58, 95% CI [1.40, 22.3], p = 0.018). In contrast, standard clinical variables, such as PD-L1 categorization (1–49% vs < 1%; p = 0.711) and Smoking History (p = 0.706), did not show statistical significance in this cohort (Supplementary Part 1).

## Discussion

Currently, clinicians have multiple first-line treatment options available for patients with aNSCLC with PD-L1 TPS < 50%. However, treatment decision-making remains challenging due to the complex nature of the immune system, its interactions with the tumor microenvironment (TME), and the absence of highly accurate biomarkers. Network analysis is a powerful tool that can reveal complex relationships and interactions within biological systems, making it invaluable for identifying predictive biomarkers and understanding the mechanisms underlying treatment responses in diseases such as cancer [22]. Previous studies have demonstrated the effectiveness of network-based approaches, such as the Network-based machine learning (NetBio) approach, in predicting immunotherapy response in cancer patients. These studies have shown that network-based models outperform traditional gene-based and tumor microenvironment-based models, emphasizing the importance of network coverage and integration of multi-omics data for robust predictions [23, 24]. Recent advances in multi-omics integration methodologies and tumor immune microenvironment profiling have provided significant insights into cancer biology and biomarker discovery. For example, optimized dynamic network biomarker approaches have been used to capture high-resolution molecular heterogeneity and transition states within tumors, underscoring the potential of network-based analyses to uncover critical functional modules and subtype-specific features across cancer types [25]. Furthermore, computational tools for systematic assessment of the tumor immune microenvironment enhance our understanding of immune cell interactions and immune phenotype landscapes across cancers, providing a broader context for interpreting associations between immune cell populations and transcriptomic signatures [26]. These studies exemplify how network-oriented frameworks and immune profiling resources can complement traditional gene-centric approaches, reinforcing the rationale for applying integrated multi-layer analyses in predictive immunotherapy research.

To our knowledge, this is the first time network analysis has been applied to aNSCLC data in a prospective manner, showcasing the novel integration of omics data for predictive biomarker discovery in immunotherapy treatment response in aNSCLC with PD-L1 TPS < 50%. By combining CIP and GEP, we gained a comprehensive understanding of the underlying mechanisms of treatment response and identified potential biomarkers.

The results of the CIP analysis revealed differences in connectivity and clustering patterns between responders and non-responder patients, suggesting that the circulating immune cell populations and their interactions play a crucial role in determining treatment response in aNSCLC. In the first evaluation of PEOPLE results by Lo Russo et al., an orthoblique principal component-based clustering approach was applied, to define a subset of clusters [4]. Each cluster score, as well as the most representative biomarker of each cluster, was tested for association with progression free survival (PFS) and overall survival (OS) by means of univariable and multivariable Cox proportional hazard models and for association with disease control rate (DCR) by means of univariable and multivariable logistic regression models. The authors found that a higher T cell and NK cell count at baseline and at the first radiologic evaluation were associated with improved PFS, DCR and OS. On the contrary, higher myeloid cell count at baseline or at the first radiologic evaluation was significantly associated with worse OS and DCR [4]. The subsequent evaluation of PEOPLE results with the multiomic LASSO analysis, selected NK cells/CD56dimCD16 + at baseline and Granulo/HLA-DRdim CD14-, non-classical CD14dim CD16 + and eosinophils (CD15 + CD16 −) after two pembrolizumab cycles, all of which were associated with a favorable PFS [10]. Our analysis confirms these findings, showing the central role of NK cells. The potential of elevated NK cells as predictive markers for the response challenges the conventional belief that immune checkpoint blockade predominantly depends on T-cell activity. Multiple pieces of evidence from both clinical and preclinical investigations underscore the pivotal role of innate immune cells in the realm of PD-1 targeted therapy [27, 28].

At the tumor level, DEA identified a limited number of DEGs, none of which showed immune-related enrichment. In contrast, DCN analysis revealed 23 hub genes and immune-associated functional modules. This discrepancy underscores a relevant biological insight: treatment response in this setting may not be driven by large magnitude changes in individual gene expression, but rather by coordinated network-level interactions among immune-related genes. Transcriptional connectivity patterns may therefore better capture functional immune readiness than isolated gene-level alterations. Using LASSO analysis, previous results within the PEOPLE trial demonstrated that five genes were identified as significant. Higher expression levels of the CD244, PTPRC, and KLRB1 genes were associated with a more favorable PFS. In contrast, higher expression levels of the IRF9 and COMP genes were linked to a poorer PFS [10]. Within our analysis, which only uses gene expression data and does not include clinical features, we also identified some relevant genes. These include CD48, the hub gene with highest degree in the DCN; its neighbors (nodes with which it has positive differential edges, and thus co-expression links significantly characterizing responders and weaker in non-responders) are enriched in the immune response. CD48 emerged as the top hub gene in the DCN, suggesting a central role in shaping the transcriptional architecture of responder tumors. CD48 is a member of the CD2 subfamily of the immunoglobulin superfamily (IgSF), which includes signaling lymphocyte activation molecule (SLAM) family proteins such as CD84, CD150, CD229, and CD244. CD48 is expressed on the surface of lymphocytes and other immune cells, including dendritic and endothelial cells, and plays a key role in immune synapse formation and bidirectional immune signaling. Through these interactions, CD48 contributes to lymphocyte activation, cytotoxicity, and cytokine production, particularly in NK cells and effector T cells [29]. Notably, CD48 acts as a ligand for CD244 (2B4), a receptor highly expressed on NK cells and CD8⁺ T lymphocytes, thereby regulating cytotoxic function and inflammatory signaling. In our study, elevated baseline NK cell levels were a distinguishing feature of responders in the circulating immune profiling analysis. The identification of CD48-centered co-expression networks in tumor tissue may therefore reflect a biologically coherent axis linking systemic NK competence with a tumor microenvironment permissive to immune synapse formation and cytotoxic lymphocyte activation. CD48-associated co-expression patterns may capture functional immune crosstalk among NK cells, cytotoxic T lymphocytes, and antigen-presenting cells, supporting sustained effector activity under PD-1 blockade, even in tumors with PD-L1 TPS < 50%. This integrated interpretation reinforces the concept that response to pembrolizumab in this setting may depend not only on tumor PD-L1 expression, but also on coordinated systemic and intratumoral immune readiness. PMS2 is another hub, but it is not enriched in terms of immune response. The PMS2 gene is a crucial component of the DNA mismatch repair (MMR) system, which is responsible for correcting errors that occur during DNA replication. From a clinical point of view, tumors with alterations in MMR genes are more likely to respond to ICIs [30]. CD45RO turns out to be a hub in the DCN but without any enrichment at the analysis of the positive connections. This gene encodes for an isoform of CD45 (PTPRC) located on memory T cells. We also investigated the PTPRC gene that was identified as the hub of the DCN, with a degree of 25. PTPRC stands for Protein Tyrosine Phosphatase Receptor Type C. It is a gene that encodes for a transmembrane protein known as CD45, expressed on various hematological cells, including T cells, B cells, and NK cells. Elevated CD45 levels could be an index of higher tumor inflammation though they are not informative regarding the type and function of tumor infiltrate since CD45 is a pan-leukocyte protein expressed on almost all immune cells [31].

These findings suggest that favorable clinical outcomes require both systemic immune competence, particularly an active NK compartment, and a tumor microenvironment enriched in leukocyte infiltration. In other words, clinical response to pembrolizumab is a systemic process and only the integrated analysis on tumor tissue and periphery can provide such evidence. As previously reported, PTPRC was identified as a relevant gene, associated with a better PFS in the LASSO analysis; also COMP gene was in this list, but it was linked to a poorer PFS. In our work, we found that COMP is a down-regulated gene, thus its expression level is lower in responders. This gene plays a role in the structural integrity of cartilage via its interaction with other extracellular matrix proteins such as the collagens and fibronectin [32]. Moreover, we extended the DCN analysis by taking a broader perspective, conducting a community detection analysis to identify modules within the network. We found one module (module 11) that is particularly enriched in terms of immune response, and it comprises 4 hub genes of the DCN. CD48 and PTPRC belong to this module. In this community analysis, we focused on identifying key hubs associated with immune system cells that were found to be "positive" in the circulating profile analysis. However, it is important to note that we were unable to identify specific gene hubs directly encoding these immune cell subsets. Moreover, to further explore systemic–local immune interactions, we performed a supplementary Spearman correlation analysis between the 31 circulating immune subsets and the expression levels of the 23 hub genes. The correlation heatmap is reported in the Supplementary Materials. Significant positive correlations were observed between CD3⁺ cells and hub genes such as CD2, CD48, and PTPRC, as well as between granulocytic-MDSCs and genes including PMS2 and EIF2B4. Interestingly, only weak or non-significant correlations were detected between baseline NK cell abundance and key hub genes such as CD48 and PTPRC. This apparent lack of strong linear associations underscores the complexity of systemic–local immune interactions. Rather than reflecting direct one-to-one relationships, these findings suggest that peripheral immune competence and intratumoral transcriptional coordination may operate through partially independent yet functionally complementary mechanisms. This finding highlights a limitation of our study and suggests that the relationships associated with the positively identified immune cells may be more complicated than initially thought.

To complement our investigation, the PSN analysis played a pivotal role in unraveling the complex interplay of GEP and CIP influencing treatment response. The networks revealed two distinct clusters of patients, shedding light on the underlying heterogeneity within the population. Notably, the identified clusters exhibited striking differences in survival outcomes. Firstly, the network constructed using only the top 23 hub genes identified in the DCN confirmed the performances obtained with all GEP variables in terms of survival prediction (Supplementary Part 1). This result underscores the importance of proper feature selection when constructing networks: the selection of key genes reduces the number of features by an order of magnitude while maintaining performance. The relatively limited predictive performance observed for the CIP-only patient similarity network (HR = 2.13, p = 0.06) suggests that peripheral immune profiling alone may not be sufficient to robustly stratify patients. Moreover, the PSN based on the 23 hub GEP variables identified clusters more effectively than the network based on CIP variables alone, as indicated by better survival HR and statistical significance in the former. However, when the similarity network analysis was performed by integrating both GEP and CIP variables, the resulting clusters showed even higher HR and statistical significance compared to networks based on either GEP or CIP variables alone. The survival disparities observed between clusters and among different similarity networks underscore the importance of integrating multiple variables, such as GEP and CIP, along with clinical factors, to more accurately predict treatment outcomes. Due to the specific clinical setting and the prospective, biomarker-driven design of the PEOPLE trial, direct validation in an independent, clinically matched aNSCLC cohort with equivalent multi-layer immunogenomic profiling is currently limited. Therefore, the stability of the SNF-based clustering was further supported by LOOCV analysis, which confirmed consistent classification accuracy and survival separation across iterations. We further evaluated whether the network-derived stratification provides prognostic information beyond standard clinical variables. In multivariable Cox regression analysis adjusting for age, smoking history, histology, PD-L1 TPS status, and ECOG performance status, the SNF-based clustering remained independently associated with overall survival, supporting the added prognostic value of the network signature beyond conventional clinicopathologic parameters.

As expected, ECOG performance status also emerged as an independent prognostic factor, consistent with its well-established role in advanced NSCLC. In contrast, other standard clinical variables, including PD-L1 stratification within the < 50% subgroup (1–49% vs < 1%) and smoking history, did not demonstrate significant prognostic discrimination in this cohort. This observation is biologically coherent with the study setting, as PD-L1 expression below the 50% threshold does not reliably capture the heterogeneity of response observed in first-line pembrolizumab-treated patients. Together, these findings suggest that the integrative network approach captures biologically relevant immune architecture that is not fully reflected by conventional clinical variables, reinforcing its potential role as a complementary stratification tool in PD-L1 < 50% aNSCLC.

However, TMB, a relevant biomarker in the context of immune checkpoint blockade, was not available for this cohort and therefore could not be included in the multivariable analysis. This represents a limitation of the present study and highlights the need for future investigations integrating additional molecular layers to further refine predictive modeling. This integrative approach enhances our ability to identify patient subgroups with shared characteristics and potential similar responses to pembrolizumab, ultimately contributing to more personalized and effective treatment strategies.

### Limitations and future perspectives

Despite the strengths of the prospective design and the integrative multi-omics framework, several limitations should be acknowledged. First, the relatively small sample size—particularly for network-based analyses performed on 38 patients and 20 responders—may increase the risk of overfitting, especially when applying complex topological methods such as differential co-expression network analysis and similarity network fusion. Although network approaches can mitigate dimensionality issues by focusing on structured interactions rather than isolated variables, the stability of identified hubs and patient clusters requires confirmation in larger independent cohorts. Second, the PEOPLE trial was conducted at a single institution within a phase II non-randomized design. This may introduce potential selection bias and limit the generalizability of our findings to broader and more heterogeneous aNSCLC populations. Third, technical variability inherent to circulating immune profiling and gene expression platforms must be considered. Differences in sample processing, sequencing depth, normalization procedures, and immune cell gating strategies could affect reproducibility across institutions. Future studies would benefit from standardized protocols and harmonized analytical pipelines to enhance cross-cohort comparability. Importantly, our findings should be interpreted as hypothesis-generating. The integrative network-based signatures identified in this study provide a biologically coherent framework linking systemic and tumor-level immune features. However, prospective validation in larger, multi-center cohorts and in independent immunotherapy datasets will be essential to assess their predictive utility in clinical practice. Future research should also explore dynamic longitudinal profiling and integration with additional molecular layers, including genomic alterations and tumor mutational burden, to refine predictive modeling strategies. At the same time, the strength of the present study lies in its prospective, biomarker-driven phase II design and its innovative use of network analysis to evaluate multiple biological layers simultaneously. This strategy provides a more comprehensive view of the immune landscape in aNSCLC with PD-L1 TPS < 50% and lays the groundwork for more personalized and effective immunotherapy approaches.

## Conclusion

This study is the first prospective drug-interventional trial investigating the association of omics biomarkers and the efficacy of immunotherapy within PD-L1 TPS < 50% aNSCLC using network analysis. Our results confirm that NK cell subsets evaluated at baseline may help identify patients who would benefit from pembrolizumab, avoiding the additional toxicity of chemotherapy. Additionally, evaluating genes such as CD48, CD45RO, and PTPRC (CD45) in tumor tissue could aid in selecting between single-agent and combination immunotherapy-chemotherapy treatments. Our patient similarity network analysis, integrating GEP and CIP data, revealed distinct patient clusters with significant survival differences, highlighting the importance of a multi-faceted approach to personalized therapy in pembrolizumab-treated patients.

## Supplementary Information

Additional file 1.

Additional file 2.

## Data Availability

The data that support the findings of this study are available on request from the corresponding author.

## Abbreviations

NSCLC: Non-small cell lung cancer
PD-L1: Programmed death-ligand 1
TPS: Tumor proportion score
aNSCLC: Advanced NSCLC
ICIs: Immune checkpoint inhibitors
CIP: Circulating immune profiling
GEP: Gene expression profiling
DEA: Differential expression analysis
DEGs: Differential expression genes
DCN: Differential co-expression networ
PSN: Patient similarity network
NK: Natural killer
HR: Hazard ratio
LOOCV: Leave one out cross validation
TME: Tumor microenvironment
PFS: Progression free survival
OS: Overall survival
DCR: Disease control rate
IgSF: Immunoglobulin superfamily
SLAM: Signaling lymphocyte activation molecules
MMR: Mismatch repair

## References

[1] Reck M, Rodríguez-Abreu D, Robinson AG, Hui R, Csőszi T, Fülöp A, et al. Pembrolizumab versus chemotherapy for PD-L1–positive non–small-cell lung cancer. N Engl J Med. 2016;375(19):1823–33. 10.1056/NEJMoa1606774. 27718847 10.1056/NEJMoa1606774

[2] Paz-Ares L, Luft A, Vicente D, Tafreshi A, Gümüş M, Mazières J, et al. Pembrolizumab plus chemotherapy for squamous non–small-cell lung cancer. N Engl J Med. 2018;379(21):2040–51. 10.1056/NEJMoa1810865. 30280635 10.1056/NEJMoa1810865

[3] Gandhi L, Rodríguez-Abreu D, Gadgeel S, Esteban E, Felip E, De Angelis F, et al. Pembrolizumab plus chemotherapy in metastatic non–small-cell lung cancer. N Engl J Med. 2018;378(22):2078–92. 10.1056/NEJMoa1801005. 29658856 10.1056/NEJMoa1801005

[4] Lo Russo G, Sgambelluri F, Prelaj A, Galli F, Manglaviti S, Bottiglieri A, et al. PEOPLE (NCT03447678), a first-line phase II pembrolizumab trial, in negative and low PD-L1 advanced NSCLC: clinical outcomes and association with circulating immune biomarkers. ESMO Open. 2022;7(6):100645. 10.1016/j.esmoop.2022.100645. 36455507 10.1016/j.esmoop.2022.100645 PMC9808469

[5] Barabási AL, Gulbahce N, Loscalzo J. Network medicine: a network-based approach to human disease. Nat Rev Genet. 2011;12(1):56–68. 10.1038/nrg2918. 21164525 10.1038/nrg2918 PMC3140052

[6] Petti M, Farina L. Network medicine for patients’ stratification: from single‐layer to multi‐omics. WIREs Mech Dis. 2023. 10.1002/wsbm.1623. 37323106 10.1002/wsbm.1623

[7] Shiravand Y, Khodadadi F, Kashani SMA, Hosseini-Fard SR, Hosseini S, Sadeghirad H, et al. Immune checkpoint inhibitors in cancer therapy. Curr Oncol. 2022;29(5):3044–60. 10.3390/curroncol29050247. 35621637 10.3390/curroncol29050247 PMC9139602

[8] Shi H, Yan KK, Ding L, Qian C, Chi H, Yu J. Network approaches for dissecting the immune system. iScience. 2020;23(8):101354. 10.1016/j.isci.2020.101354. 32717640 10.1016/j.isci.2020.101354 PMC7390880

[9] Alfano C, Farina L, Petti M. Networks as biomarkers: uses and purposes. Genes. 2023;14(2):429. 10.3390/genes14020429. 36833356 10.3390/genes14020429 PMC9956930

[10] Lo Russo G, Prelaj A, Dolezal J, Beninato T, Agnelli L, Triulzi T, et al. PEOPLE (NTC03447678), a phase II trial to test pembrolizumab as first-line treatment in patients with advanced NSCLC with PD-L1 50%: a multiomics analysis. J Immunother Cancer. 2023;11(6):e006833. 10.1136/jitc-2023-006833. 37286305 10.1136/jitc-2023-006833 PMC10254948

[11] Love MI, Huber W, Anders S. Moderated estimation of fold change and dispersion for RNA-seq data with DESeq2. Genome Biol. 2014;15(12):550. 10.1186/s13059-014-0550-8. 25516281 10.1186/s13059-014-0550-8 PMC4302049

[12] Fukushima A. DiffCorr: an R package to analyze and visualize differential correlations in biological networks. Gene. 2013;518(1):209–14. 10.1016/j.gene.2012.11.028. 23246976 10.1016/j.gene.2012.11.028

[13] Blondel VD, Guillaume JL, Lambiotte R, Lefebvre E. Fast unfolding of communities in large networks. J Stat Mech Theory Exp. 2008;2008(10):P10008. 10.1088/1742-5468/2008/10/P10008.

[14] Kanehisa M, Furumichi M, Sato Y, Matsuura Y, Ishiguro-Watanabe M. KEGG: biological systems database as a model of the real world. Nucleic Acids Res. 2025;53(D1):D672–7. 10.1093/nar/gkae909. 39417505 10.1093/nar/gkae909 PMC11701520

[15] Kanehisa M. Toward understanding the origin and evolution of cellular organisms. Protein Sci. 2019;28(11):1947–51. 10.1002/pro.3715. 31441146 10.1002/pro.3715 PMC6798127

[16] Kanehisa M. KEGG: Kyoto Encyclopedia of Genes and Genomes. Nucleic Acids Res. 2000;28(1):27–30. 10.1093/nar/28.1.27. 10592173 10.1093/nar/28.1.27 PMC102409

[17] Wang B, Mezlini AM, Demir F, Fiume M, Tu Z, Brudno M, et al. Similarity network fusion for aggregating data types on a genomic scale. Nat Methods. 2014;11(3):333–7. 10.1038/nmeth.2810. 24464287 10.1038/nmeth.2810

[18] Zhang J, Li Y, Dai W, Tang F, Wang L, Wang Z, et al. Molecular classification reveals the sensitivity of lung adenocarcinoma to radiotherapy and immunotherapy: multi-omics clustering based on similarity network fusion. Cancer Immunol Immunother. 2024;73(4):71. 10.1007/s00262-024-03657-x. 38430394 10.1007/s00262-024-03657-x PMC10908647

[19] Wang C, Lue W, Kaalia R, Kumar P, Rajapakse JC. Network-based integration of multi-omics data for clinical outcome prediction in neuroblastoma. Sci Rep. 2022;12(1):15425. 10.1038/s41598-022-19019-5. 36104347 10.1038/s41598-022-19019-5 PMC9475034

[20] Bhalla S, Melnekoff DT, Aleman A, Leshchenko V, Restrepo P, Keats J, et al. Patient similarity network of newly diagnosed multiple myeloma identifies patient subgroups with distinct genetic features and clinical implications. Sci Adv. 2021. 10.1126/sciadv.abg9551. 34788103 10.1126/sciadv.abg9551 PMC8598000

[21] Cavalli FMG, Remke M, Rampasek L, Peacock J, Shih DJH, Luu B, et al. Intertumoral heterogeneity within medulloblastoma subgroups. Cancer Cell. 2017;31(6):737-754.e6. 10.1016/j.ccell.2017.05.005. 28609654 10.1016/j.ccell.2017.05.005 PMC6163053

[22] Kong J, Ha D, Lee J, Kim I, Park M, Im SH, et al. Network-based machine learning approach to predict immunotherapy response in cancer patients. Nat Commun. 2022;13(1):3703. 10.1038/s41467-022-31535-6. 35764641 10.1038/s41467-022-31535-6 PMC9240063

[23] Barry KC, Hsu J, Broz ML, Cueto FJ, Binnewies M, Combes AJ, et al. A natural killer–dendritic cell axis defines checkpoint therapy–responsive tumor microenvironments. Nat Med. 2018;24(8):1178–91. 10.1038/s41591-018-0085-8. 29942093 10.1038/s41591-018-0085-8 PMC6475503

[24] Filetti M, Occhipinti M, Cirillo A, Scirocchi F, Ugolini A, Giusti R, et al. Exploring genomic biomarkers for Pembrolizumab response: a real-world approach and patient similarity network analysis reveal DNA response and repair gene mutations as a signature. Cancers (Basel). 2024;16(23):3955. 10.3390/cancers16233955. 39682144 10.3390/cancers16233955 PMC11639826

[25] Zhang H, Zhang G, Xu P, Yu F, Li L, Huang R, et al. Optimized dynamic network biomarker deciphers a high‐resolution heterogeneity within thyroid cancer molecular subtypes. Med Res. 2025;1(1):10–31. 10.1002/mdr2.70004.

[26] Liu L, Xie Y, Yang H, Lin A, Dong M, Wang H, et al. HPVTIMER: a shiny web application for tumor immune estimation in human papillomavirus‐associated cancers. iMeta. 2023. 10.1002/imt2.130. 38867938 10.1002/imt2.130 PMC10989930

[27] Dong W, Wu X, Ma S, Wang Y, Nalin AP, Zhu Z, et al. The mechanism of anti–PD-L1 antibody efficacy against PD-L1–negative tumors identifies NK cells expressing PD-L1 as a cytolytic effector. Cancer Discov. 2019;9(10):1422–37. 10.1158/2159-8290.CD-18-1259. 31340937 10.1158/2159-8290.CD-18-1259 PMC7253691

[28] Hsu J, Hodgins JJ, Marathe M, Nicolai CJ, Bourgeois-Daigneault MC, Trevino TN, et al. Contribution of NK cells to immunotherapy mediated by PD-1/PD-L1 blockade. J Clin Invest. 2018;128(10):4654–68. 10.1172/JCI99317. 30198904 10.1172/JCI99317 PMC6159991

[29] https://www.ncbi.nlm.nih.gov/gene?Db=gene&Cmd=ShowDetailView&TermToSearch=962. NCBI Gene Database.

[30] Ricciuti B, Recondo G, Spurr LF, Li YY, Lamberti G, Venkatraman D, et al. Impact of DNA damage response and repair (DDR) gene mutations on efficacy of PD-(L)1 immune checkpoint inhibition in non–small cell lung cancer. Clin Cancer Res. 2020;26(15):4135–42. 10.1158/1078-0432.CCR-19-3529. 32332016 10.1158/1078-0432.CCR-19-3529

[31] Al Barashdi MA, Ali A, McMullin MF, Mills K. Protein tyrosine phosphatase receptor type C (PTPRC or CD45). J Clin Pathol. 2021;74(9):548–52. 10.1136/jclinpath-2020-206927. 34039664 10.1136/jclinpath-2020-206927 PMC8380896

[32] Wisłowska M, Jabłońska B. Serum cartilage oligomeric matrix protein (COMP) in rheumatoid arthritis and knee osteoarthritis. Clin Rheumatol. 2005;24(3):278–84. 10.1007/s10067-004-1000-x. 15940561 10.1007/s10067-004-1000-x

