# Supplementary material for: Network analysis predicts pembrolizumab response in advanced NSCLC with PD-L1 < 50%
Source: Cancer Cell Int. 2026 Mar 26;26:190. doi: 10.1186/s12935-026-04268-5 (PMC13147633; doi:10.1186/s12935-026-04268-5)
Supplement: Supplementary file 1 — Additional file 1. [file 12935_2026_4268_MOESM1_ESM.docx]

## **Supplementary Part 1 - Results**

**Patients’ characteristics and survival analysis**

From May 31, 2018, to October 07, 2020, 87 treatment naïve patients with aNSCLC diagnosis were screened. Data lock was set on July 22, 2021. Among the 87 patients screened, 17 were declared screening failure and 65 were enrolled and treated. The median age was 70 years (47–87), with 44 (68%) men and 21 (32%) women. ECOG PS was 0 in 23 (35.4%), 1 in 30 (46.2%), and 2 in 12 (18.5%) patients. Most patients (50, 77%) had an adenocarcinoma histology while 10 (15%) patients had a squamous histology and 5 (8%) other types of histology. PD-L1 TPS expression was 1–49% in 47 (73%) patients and 0 in 18 (27%) patients. Twenty-eight (43.1%) patients received antibiotics during study treatment. Demographic and clinical baseline patient characteristics are reported in the Table 11. At the time of analysis, with a median follow-up of 26.4 months (mo), 51 (78%) patients experienced progression and 46 (70%) died. The median PFS was 2.9 mo (95% CI 1.8 to 5.8) and the median OS was 12.1 mo (95% CI 9.0 to 20.2). Seven patients experienced an early death (before the first radiological evaluation), while 58 patients underwent at least one radiological evaluation. The ORR was 24.1%, DCR was 53.4% and the median DoR was 14.5 mo (95%CI 8.4 to 24.9).

|  | All patients N = 65 | Patients with biomarker profile  at baseline N = 57 | Patients with biomarker profile  at baseline and at first disease evaluation N = 46 |
| --- | --- | --- | --- |
| **Age (years)**  Median (Q1-Q3) | 70.9 (63.7-77.1) | 70.9 (63.7-75.6) | 69.3 (63.7-75.0) |
| **Female sex, n (%)** | 21 (32.3) | 20 (35.1) | 17 (37.0) |
| **Race, n (%)**  Asian  Black or African American White | 1 (1.5)  1 (1.5)  63 (96.9) | 0 (0.0)  0 (0.0)  57 (100.0) | 0 (0.0)  0 (0.0)  46 (100.0) |
| **Smoking habits, n (%)a**  Current Former Never | 15 (23.1)  42 (64.6)  8 (12.3) | 13 (22.8)  38 (66.7)  6 (10.5) | 12 (26.1)  28 (60.9)  6 (13.0) |
| **ECOG performance status, n (%)**  0  1  2 | 23 (35.4)  30 (46.2)  12 (18.5) | 22 (38.6)  25 (43.9)  10 (17.5) | 20 (43.5)  20 (43.5)  6 (13.0) |
| **Histology, n (%)**  Non-squamous Squamous | 54 (83.1)  11 (16.9) | 49 (86.0)  8 (14.0) | 42 (91.3)  4 (8.7) |
| **Number of metastatic sites, n (%)**  0-2  >2 | 47 (72.3)  18 (27.7) | 44 (77.2)  13 (22.8) | 38 (82.6)  8 (17.4) |
| **Liver metastases, n (%)** | 5 (7.7) | 3 (5.3) | 3 (6.5) |
| **Brain metastases, n (%)** | 10 (15.4) | 8 (14.0) | 8 (17.4) |
| **Bone metastases, n (%)** | 10 (15.4) | 8 (14.0) | 8 (17.4) |
| **Clinical staging at study entry, n (%)**  IIIB IV | 3 (4.6)  62 (95.4) | 3 (5.3)  54 (94.7) | 2 (4.3)  44 (95.7) |
| **Categorical PD-L1 status, n (%)**  0  1-49 | 19 (29.2)  46 (70.8) | 17 (29.8)  40 (70.2) | 14 (30.4)  32 (69.6) |
| **Continuous PD-L1 status**  Mean (SD) Median (Q1-Q3) Missing | 12.1 (16.7)  2.3 (0.0-20.0)  3 | 11.2 (15.6)  2.3 (0.0-20.0)  3 | 9.6 (13.9)  2.0 (0.0-10.0)  1 |
| **Neo/adjuvant chemotherapy, n (%)**  No Yes | 62 (95.4)  3 (4.6) | 55 (96.5)  2 (3.5) | 45 (97.8)  1 (2.2) |

Supplementary Table 1. Patients’ demographic and clinical characteristics at baseline


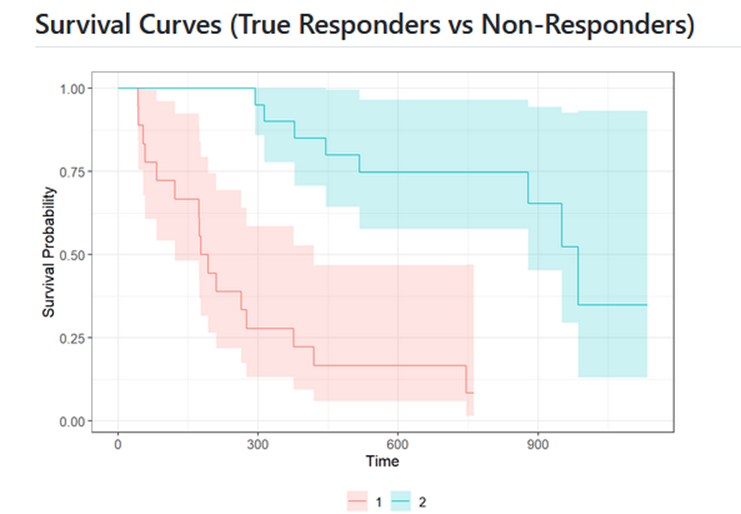


Supplementary Figure 1. Kaplan-Meier real-world survival curves

**Differential Expression Analysis**

A subset of 14 genes met these criteria, 8 up-regulated and 6 down-regulated, as shown in Supplementary Figure 1. No enriched term was found at enrichment analysis.


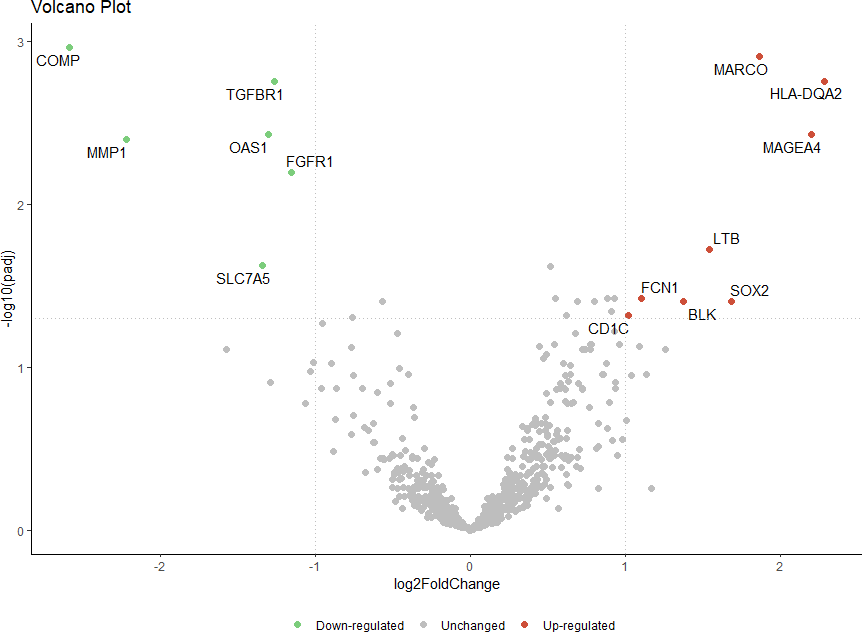


Supplementary Figure 2. Volcano Plot of differentially expressed genes.

| **Gene** | **baseMean** | **log2FoldChange** | **lfcSE** | **stat** | **pvalue** | **padj** | **expression** |
| --- | --- | --- | --- | --- | --- | --- | --- |
| COMP | 826.71793 | -2.586946 | 0.5370844 | -4.816647 | 0.0000015 | 0.0010978 | Downregulated |
| MARCO | 810.57292 | 1.867745 | 0.4015382 | 4.651476 | 0.0000033 | 0.0012392 | Up regulated |
| HLA-DQA2 | 145.54255 | 2.287729 | 0.5162102 | 4.431779 | 0.0000093 | 0.0017570 | Up regulated |
| TGFBR1 | 1514.43147 | -1.266021 | 0.2849144 | -4.443515 | 0.0000089 | 0.0017570 | Downregulated |
| OAS1 | 1371.36089 | -1.302983 | 0.3102452 | -4.199849 | 0.0000267 | 0.0037262 | Downregulated |
| MAGEA4 | 101.39310 | 2.199960 | 0.5268703 | 4.175525 | 0.0000297 | 0.0037262 | Up regulated |
| MMP1 | 1321.43695 | -2.217866 | 0.5377547 | -4.124308 | 0.0000372 | 0.0039947 | Downregulated |
| FGFR1 | 472.74387 | -1.152212 | 0.2892436 | -3.983534 | 0.0000679 | 0.0063824 | Downregulated |
| LTB | 530.46164 | 1.540088 | 0.4176544 | 3.687470 | 0.0002265 | 0.0189249 | Up regulated |
| SLC7A5 | 660.92617 | -1.342197 | 0.3726268 | -3.601987 | 0.0003158 | 0.0237477 | Downregulated |
| FCN1 | 257.19163 | 1.107087 | 0.3249620 | 3.406820 | 0.0006572 | 0.0379903 | Up regulated |
| SOX2 | 393.93166 | 1.683317 | 0.5095532 | 3.303516 | 0.0009548 | 0.0396858 | Up regulated |
| BLK | 56.80966 | 1.377780 | 0.4204512 | 3.276909 | 0.0010495 | 0.0396858 | Up regulated |
| CD1C | 68.63501 | 1.021145 | 0.3204178 | 3.186918 | 0.0014380 | 0.0482961 | Up regulated |

Supplementary Table 2. Differentially Expressed Genes.

**Co-expression network and differential co-expression network (DCN) of gene expression profiling (GEP)**

**A**

| CD48 |
| --- |
| ITGAL |
| CD3D |
| PTPRC |
| PFKM |
| PMS2 |
| BIRC5 |
| ERCC3 |
| GIMAP4 |
| ZAP70 |
| CD2 |
| CD45RB |
| PIK3CD |
| PIK3R5 |
| CDC20 |
| HELLS |
| IL16 |
| CD45RO |
| CDK2 |
| IL2RB |
| ITGA4 |
| JAK3 |
| UBE2T |

**B**

| CSF1R |
| --- |
| NOTCH2 |
| COL11A1 |
| COL5A1 |
| DAB2 |
| EIF2B4 |
| FCGR2A |
| PGPEP1 |
| CDH11 |
| ZEB2 |
| TNFRSF10D |
| AXIN1 |
| CD45RO |
| LAIR1 |
| SERPINH1 |
| INHBA |
| OLFML2B |
| FCGR1A |

Supplementary Table 3. A: Hubs of responders co-expression networks. B: Hubs of non-responders co-expression networks.


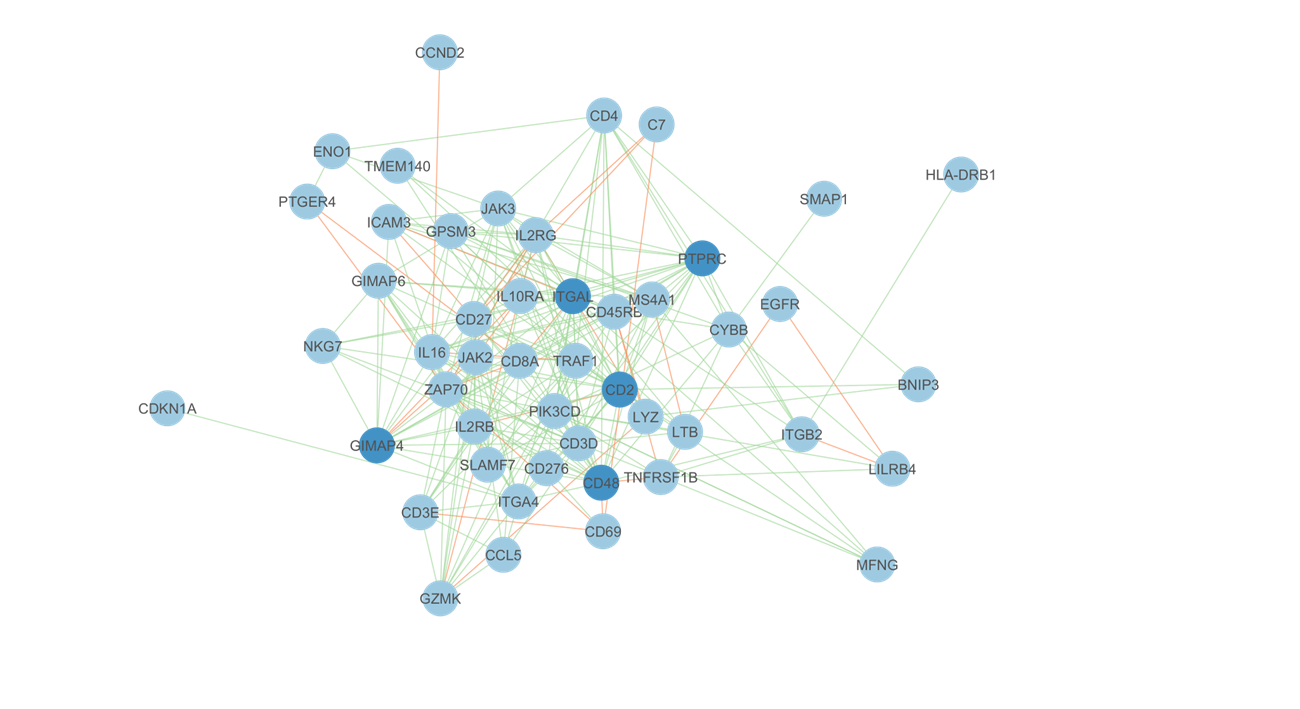


Supplementary Figure 3. Module 11. Hub nodes of the DCN highlighted in dark blue.


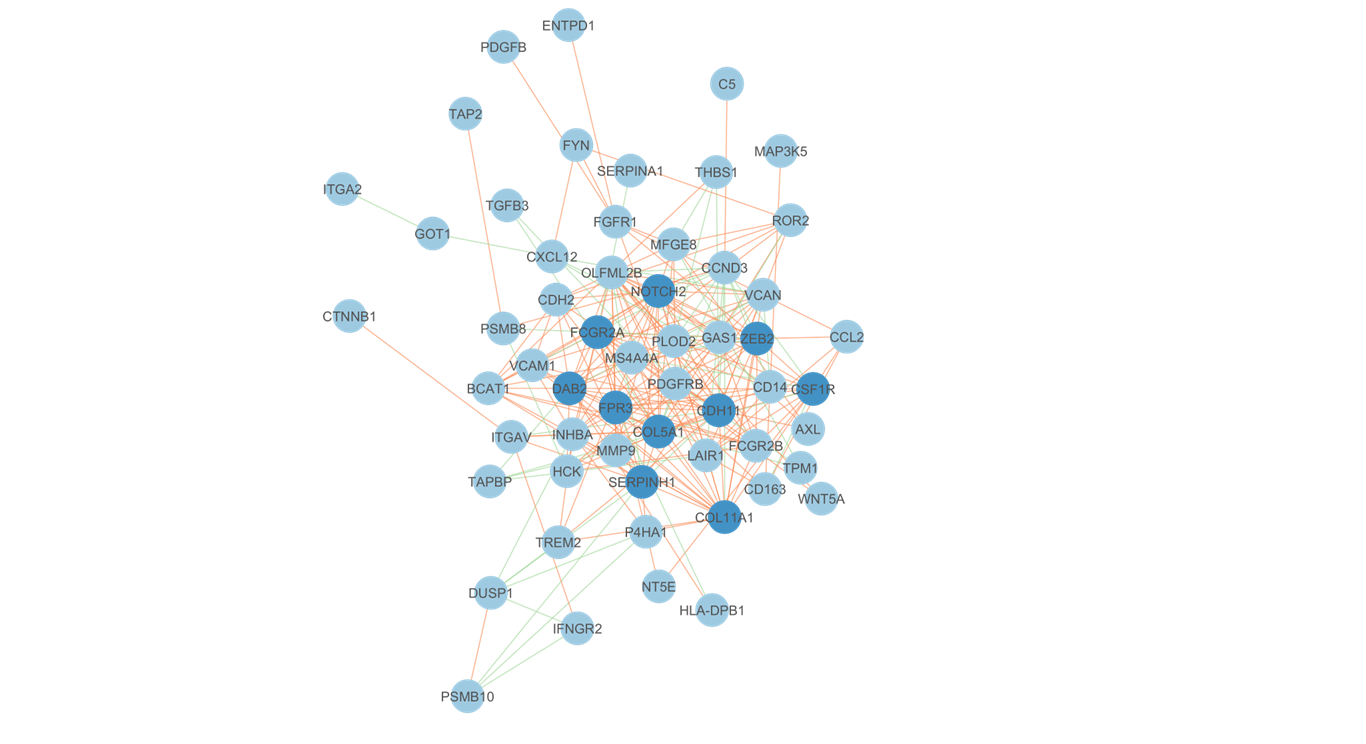


Supplementary Figure 4. Module 10. Hub nodes of the DCN highlighted in dark blue.

**A**

| BNIP3 |
| --- |
| C7 |
| CCL5 |
| CCND2 |
| CD2 |
| CD27 |
| CD276 |
| CD3D |
| CD3E |
| CD4 |
| CD45RB |
| CD48 |
| CD69 |
| CD8A |
| CDKN1A |
| CYBB |
| EGFR |
| ENO1 |
| GIMAP4 |
| GIMAP6 |
| GPSM3 |
| GZMK |
| HLA-DRB1 |
| ICAM3 |
| IL10RA |
| IL16 |
| IL2RB |
| IL2RG |
| ITGA4 |
| ITGAL |
| ITGB2 |
| JAK2 |
| JAK3 |
| LILRB4 |
| LTB |
| LYZ |
| MFNG |
| MS4A1 |
| NKG7 |
| PIK3CD |
| PTGER4 |
| PTPRC |
| SLAMF7 |
| SMAP1 |
| TMEM140 |
| TNFRSF1B |
| TRAF1 |
| ZAP70 |

**B**

| AXL |
| --- |
| BCAT1 |
| C5 |
| CCL2 |
| CCND3 |
| CD14 |
| CD163 |
| CDH11 |
| CDH2 |
| COL11A1 |
| COL5A1 |
| CSF1R |
| CTNNB1 |
| CXCL12 |
| DAB2 |
| DUSP1 |
| ENTPD1 |
| FCGR2A |
| FCGR2B |
| FCGR3A/B |
| FGFR1 |
| FPR3 |
| FYN |
| GAS1 |
| GOT1 |
| HCK |
| HLA-DPB1 |
| IFNGR2 |
| INHBA |
| ITGA2 |
| ITGAV |
| LAIR1 |
| MAP3K5 |
| MFGE8 |
| MMP9 |
| MS4A4A |
| NOTCH2 |
| NT5E |
| OLFML2B |
| P4HA1 |
| PDGFB |
| PDGFRB |
| PLOD2 |
| PSMB10 |
| PSMB8 |
| ROR2 |
| SERPINA1 |
| SERPINH1 |
| TAP2 |
| TAPBP |
| TGFB3 |
| THBS1 |
| TPM1 |
| TREM2 |
| VCAM1 |
| VCAN |
| WNT5A |
| ZEB2 |

Supplementary Table 4. A: Hubs genes in Module 11. B: Hubs genes in Module 10.

**Stratification of patient survival based on CIP and GEP integration**

**Patient Similarity Networks**

|  |
| --- |
| 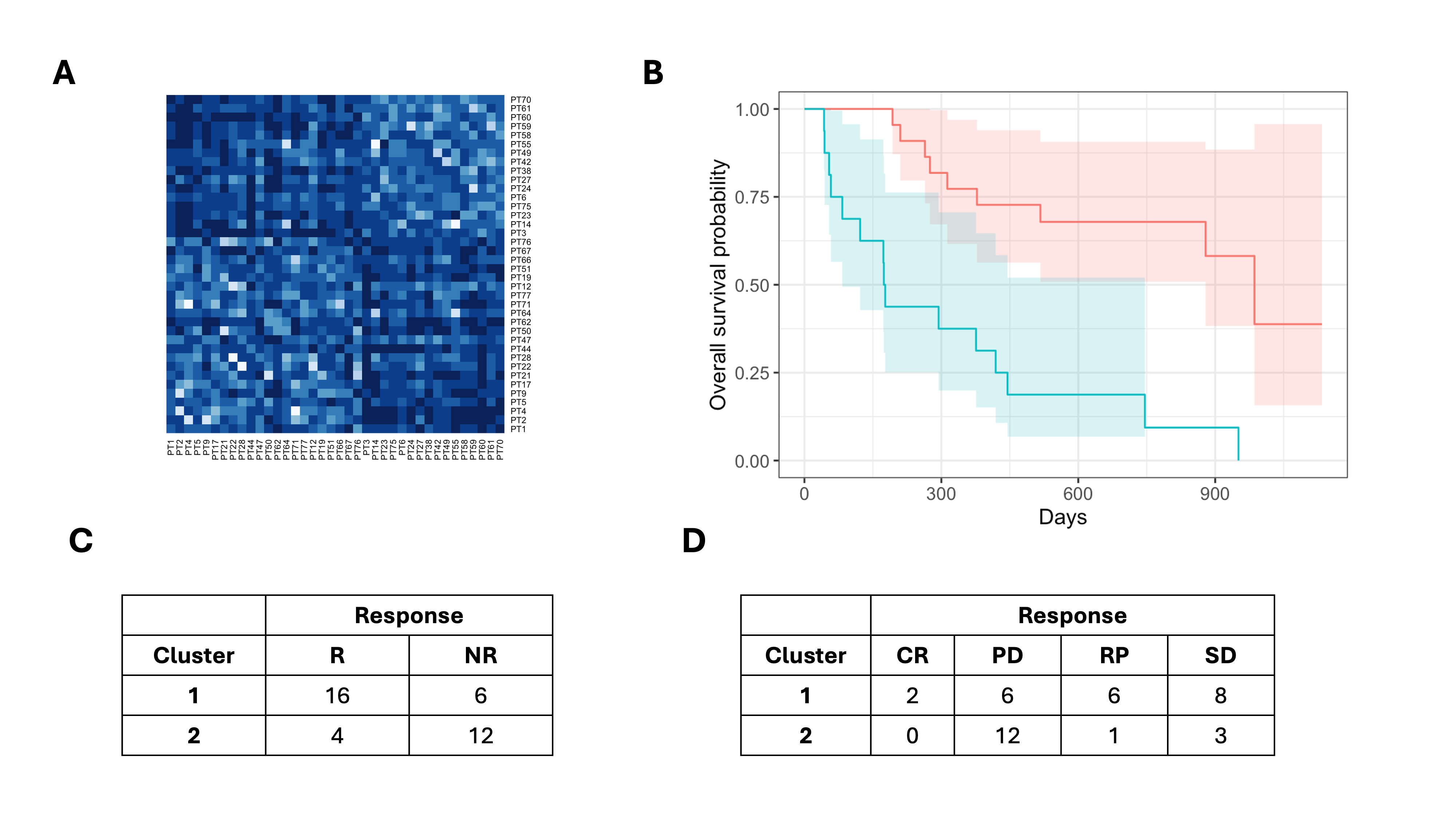 |

**Supplementary Figure 5. Patient Similarity Network of 38 patients, using all DCN genes. A: PSN for 38 patients, using squared eucledian distance. B*:* Kaplan-Meier survival analysis for Cluster 1 (red) and Cluster 2 (cyan). Log-rank test *p*-value < 0.001. Cox regression model: Hazard Ratio (HR) = 5.27, 95% CI [2.20, 12.60], *p*-value < 0.001. C: Confusion matrix comparing PSN clusters with true response labels (NR = Non-Responders, R = Responders). Accuracy = 0.74. D: Confusion matrix comparing PSN clusters with true response labels (CR = Complete Response, PD = Progressive Disease, RP = Partial Response, SD = Stable Disease).**

|  |
| --- |
|  |


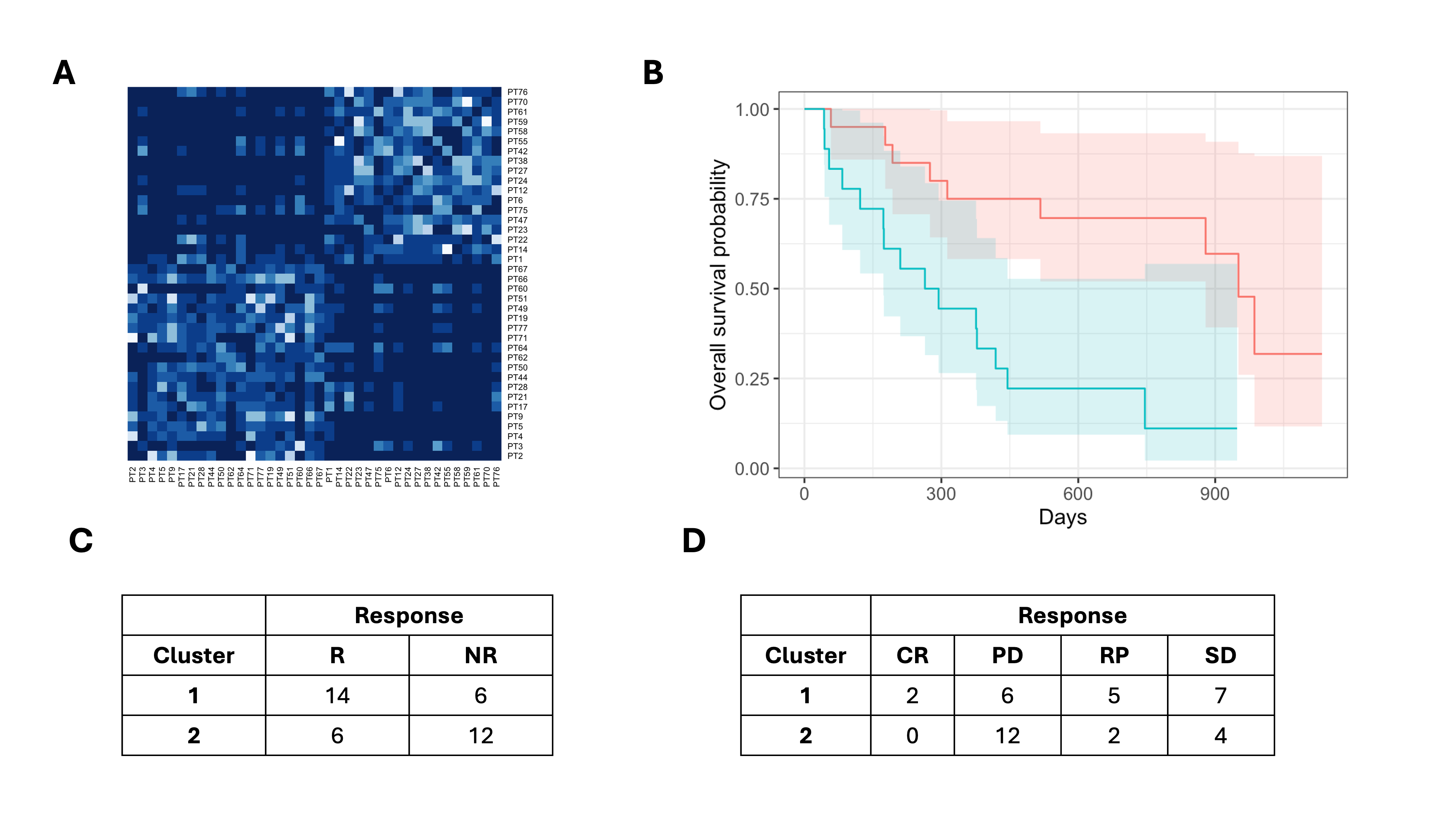


**Supplementary Figure 6. Patient Similarity Network of 38 patients, using first 10 DCN hubs. A: PSN for 38 patients, using squared eucledian distance. B*:* Kaplan-Meier survival analysis for Cluster 1 (red) and Cluster 2 (cyan). Log-rank test *p*-value = 0.001. Cox regression model: Hazard Ratio (HR) = 4.20, 95% CI [1.67, 10.50], *p*-value = 0.002. C: Confusion matrix comparing PSN clusters with true response labels (NR = Non-Responders, R = Responders). Accuracy = 0.68. D: Confusion matrix comparing PSN clusters with true response labels (CR = Complete Response, PD = Progressive Disease, RP = Partial Response, SD = Stable Disease).**

|  |
| --- |


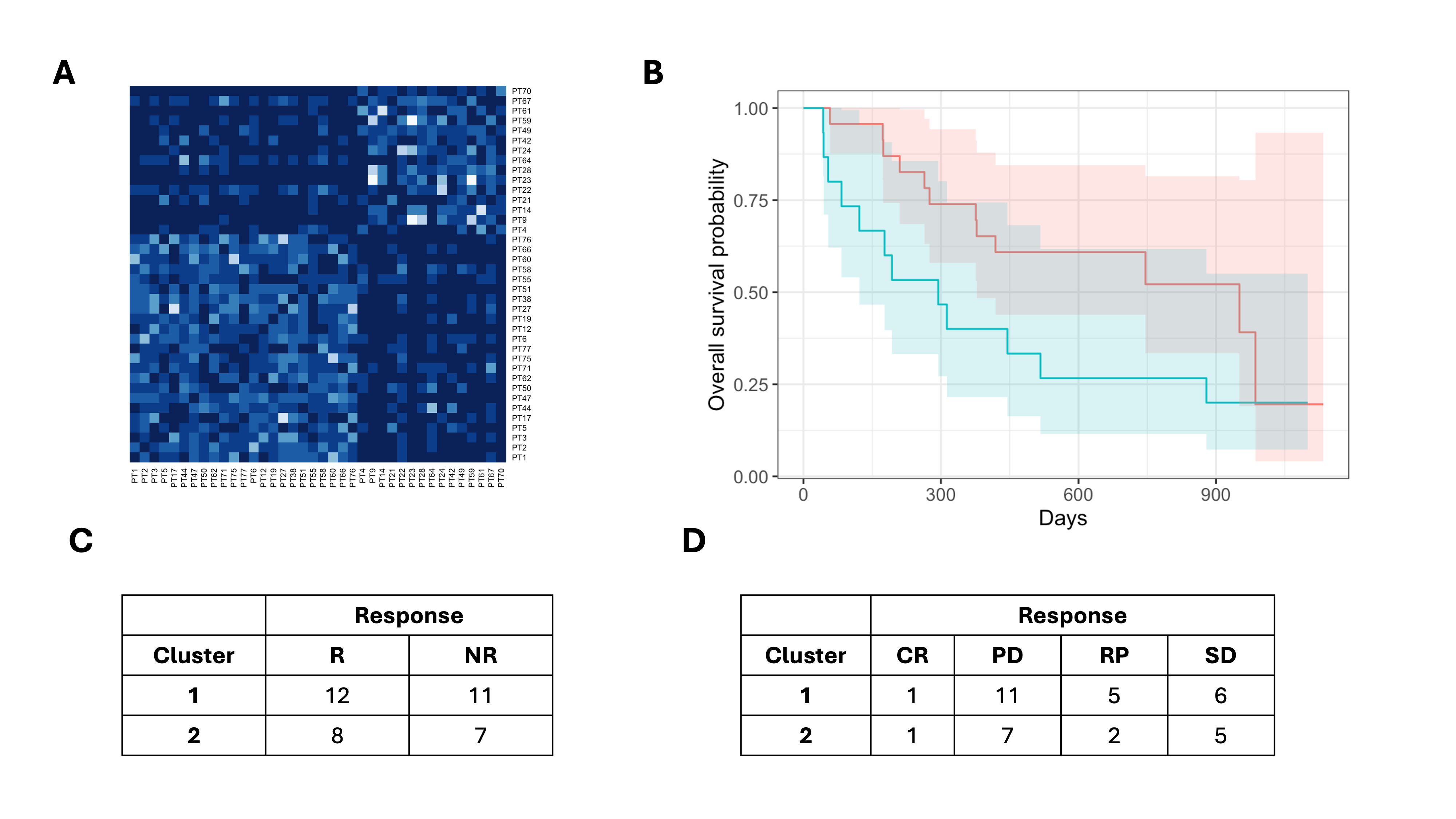


**Supplementary Figure 7. Patient Similarity Network of 38 patients, using all circulating immune subsets. A: PSN for 38 patients. B*:* Kaplan-Meier survival analysis for Cluster 1 (red) and Cluster 2 (cyan). Log-rank test *p*-value = 0.06. Cox regression model: Hazard Ratio (HR) = 2.13, 95% CI [0.95, 4.76], *p*-value = 0.067. C: Confusion matrix comparing PSN clusters with true response labels (NR = Non-Responders, R = Responders). Accuracy = 0.5. D: Confusion matrix comparing PSN clusters with true response labels (CR = Complete Response, PD = Progressive Disease, RP = Partial Response, SD = Stable Disease).**

| **Variable** | **Median (IQR) Cluster 1** | **Median (IQR) Cluster 2** | **P-Value** | **Sig.** | **FDR** |
| --- | --- | --- | --- | --- | --- |
| COL11A1 | 10.82 (10.25, 12.41) | 6.86 (5.49, 8.22) | < 0.001 | *** | < 0.001 |
| CDH11 | 12.39 (11.90, 12.67) | 11.00 (10.63, 11.05) | < 0.001 | *** | < 0.001 |
| COL5A1 | 12.61 (12.13, 13.03) | 10.63 (10.22, 11.05) | < 0.001 | *** | < 0.001 |
| SERPINH1 | 10.64 (10.20, 10.73) | 9.34 (8.87, 9.84) | < 0.001 | *** | < 0.001 |
| FCGR2A | 11.24 (10.68, 11.52) | 10.17 (9.91, 10.83) | < 0.001 | *** | 0.004 |
| CD48 | 7.40 (6.89, 7.81) | 8.39 (7.98, 9.15) | 0.002 | ** | 0.008 |
| DAB2 | 9.73 (9.53, 9.88) | 9.15 (8.64, 9.64) | 0.003 | ** | 0.010 |
| ITGAL | 7.81 (7.35, 8.61) | 8.59 (8.30, 9.04) | 0.003 | ** | 0.010 |
| ZEB2 | 10.57 (10.34, 10.74) | 9.95 (9.72, 10.45) | 0.004 | ** | 0.011 |
| CSF1R | 10.68 (10.28, 11.14) | 9.97 (9.34, 10.65) | 0.009 | ** | 0.020 |
| NOTCH2 | 10.61 (10.52, 10.86) | 10.39 (9.87, 10.59) | 0.010 | ** | 0.020 |
| PTPRC | 8.06 (7.84, 8.31) | 8.63 (8.25, 9.32) | 0.012 | * | 0.024 |
| FPR3 | 9.38 (9.09, 9.66) | 9.09 (8.74, 9.28) | 0.038 | * | 0.068 |
| PGPEP1 | 7.09 (6.78, 7.39) | 7.52 (7.14, 8.52) | 0.048 | * | 0.078 |
| CD2 | 6.98 (6.70, 7.23) | 7.40 (6.97, 7.86) | 0.055 |  | 0.084 |
| PTPN11 | 10.91 (10.60, 11.04) | 10.57 (10.31, 10.95) | 0.087 |  | 0.125 |
| ERCC3 | 9.86 (9.67, 10.09) | 9.58 (9.12, 9.91) | 0.093 |  | 0.126 |
| CDC20 | 7.44 (6.74, 8.20) | 6.83 (6.01, 7.82) | 0.144 |  | 0.184 |
| EIF2B4 | 8.01 (7.81, 8.39) | 7.89 (7.64, 8.18) | 0.438 |  | 0.531 |
| CD45RO | 9.40 (9.06, 9.70) | 9.37 (9.16, 10.12) | 0.534 |  | 0.606 |
| PMS2 | 8.71 (8.04, 8.87) | 8.28 (7.77, 8.89) | 0.553 |  | 0.606 |
| GIMAP4 | 9.87 (9.54, 10.07) | 9.79 (9.63, 10.28) | 0.661 |  | 0.691 |
| UBE2T | 7.11 (6.73, 8.24) | 7.28 (6.60, 8.39) | 0.965 |  | 0.965 |

**Supplementary Table 5. Comparison of expression levels for the 23 hub genes between SNF Cluster 1 (Responder-like) and Cluster 2 (Non-Responder-like). Data are presented as Median (Interquartile Range, IQR) of normalized gene counts. P-values were calculated using the Mann-Whitney U test. Significance level: *** p < 0.001, ** p < 0.01, * p < 0.05.**

| **Variable** | **Median (IQR) Cluster 1** | **Median (IQR) Cluster 2** | **P-Value** | **Sig.** | **FDR** |
| --- | --- | --- | --- | --- | --- |
| CD14+ | -0.65 (-0.98, -0.47) | 0.61 (0.26, 1.38) | < 0.001 | *** | < 0.001 |
| Classical CD14+CD16- | -0.73 (-0.96, -0.50) | 0.77 (0.27, 1.44) | < 0.001 | *** | < 0.001 |
| CD14+/HLA-DR+ | -0.61 (-1.11, -0.38) | 0.63 (0.35, 0.91) | < 0.001 | *** | < 0.001 |
| Classical CD14+CD16-/HLA-DR+ | -0.75 (-1.14, -0.40) | 0.62 (0.47, 0.98) | < 0.001 | *** | < 0.001 |
| Granulo-/HLA-DRdim CD14+ | -0.69 (-0.94, -0.48) | 0.81 (0.07, 1.85) | < 0.001 | *** | < 0.001 |
| m-MDSCs (HLA-DRLo, CD33+, CD15-, CD14+) | -0.66 (-0.81, -0.41) | 0.95 (0.23, 1.83) | < 0.001 | *** | < 0.001 |
| CD15hi | -0.51 (-0.80, -0.27) | 0.72 (0.34, 1.54) | < 0.001 | *** | < 0.001 |
| Neutrophils (CD15+CD16+) | -0.49 (-0.78, -0.27) | 0.77 (0.31, 1.51) | < 0.001 | *** | < 0.001 |
| g-MDSCs (HLA-DRLo, CD33+, CD15+, CD14-) | -0.42 (-0.53, -0.38) | 0.17 (-0.25, 1.16) | < 0.001 | *** | < 0.001 |
| Intermediate CD14+CD16+ | -0.42 (-0.76, -0.21) | 0.11 (-0.53, 1.45) | 0.017 | * | 0.053 |
| NK cells /CD56brCD16-/HLA-DR+ | -0.39 (-0.54, -0.16) | -0.20 (-0.32, 0.30) | 0.078 |  | 0.201 |
| Intermediate CD14+CD16+/HLA-DR+++ | -0.46 (-0.75, -0.18) | 0.01 (-0.60, 1.46) | 0.078 |  | 0.201 |
| NK T-Like cells | -0.26 (-0.52, 0.37) | -0.47 (-0.68, -0.21) | 0.213 |  | 0.507 |
| NK cells /CD56dimCD16-/HLA-DR+ | -0.28 (-0.69, -0.04) | -0.16 (-0.63, 0.47) | 0.286 |  | 0.634 |
| NK cells (all CD3- CD56+) | -0.38 (-0.81, 0.13) | -0.19 (-0.56, 0.54) | 0.344 |  | 0.710 |
| NK T-Like cells/HLA-DR+ | -0.03 (-0.68, 0.52) | -0.48 (-0.63, -0.08) | 0.391 |  | 0.743 |
| Eosinophils (CD15+CD16-) | -0.41 (-0.85, 0.24) | 0.34 (-0.64, 0.84) | 0.408 |  | 0.743 |
| NK cells /HLA-DR+ | -0.54 (-0.78, 0.23) | -0.33 (-0.78, 0.81) | 0.478 |  | 0.800 |
| NK cells /CD56dimCD16+ | -0.41 (-0.75, 0.17) | -0.20 (-0.61, 0.46) | 0.497 |  | 0.800 |
| Non-classical CD14dimCD16+ | -0.10 (-0.58, 0.52) | -0.32 (-0.99, 0.51) | 0.516 |  | 0.800 |
| Non-classical CD14dimCD16+/HLA-DR++ | -0.09 (-0.69, 0.24) | -0.27 (-0.96, 0.46) | 0.595 |  | 0.862 |
| NK cells /CD56dimCD16+/HLA-DR+ | -0.47 (-0.70, 0.34) | -0.41 (-0.70, 0.41) | 0.637 |  | 0.862 |
| CD19+ | -0.20 (-0.46, 0.18) | -0.28 (-0.39, 0.03) | 0.680 |  | 0.862 |
| CD19+/HLA-DR+ | -0.20 (-0.46, 0.18) | -0.27 (-0.38, 0.03) | 0.680 |  | 0.862 |
| NK cells /CD56brCD16- | -0.23 (-0.47, 0.06) | -0.18 (-0.42, 0.14) | 0.723 |  | 0.862 |

**Supplementary Table 6. Comparison of the 31 circulating immune cell subsets between SNF Cluster 1 (Responder-like) and Cluster 2 (Non-Responder-like). Data are presented as Median (Interquartile Range, IQR) of normalized gene counts. P-values were calculated using the Mann-Whitney U test. Significance level: *** p < 0.001, ** p < 0.01, * p < 0.05.**


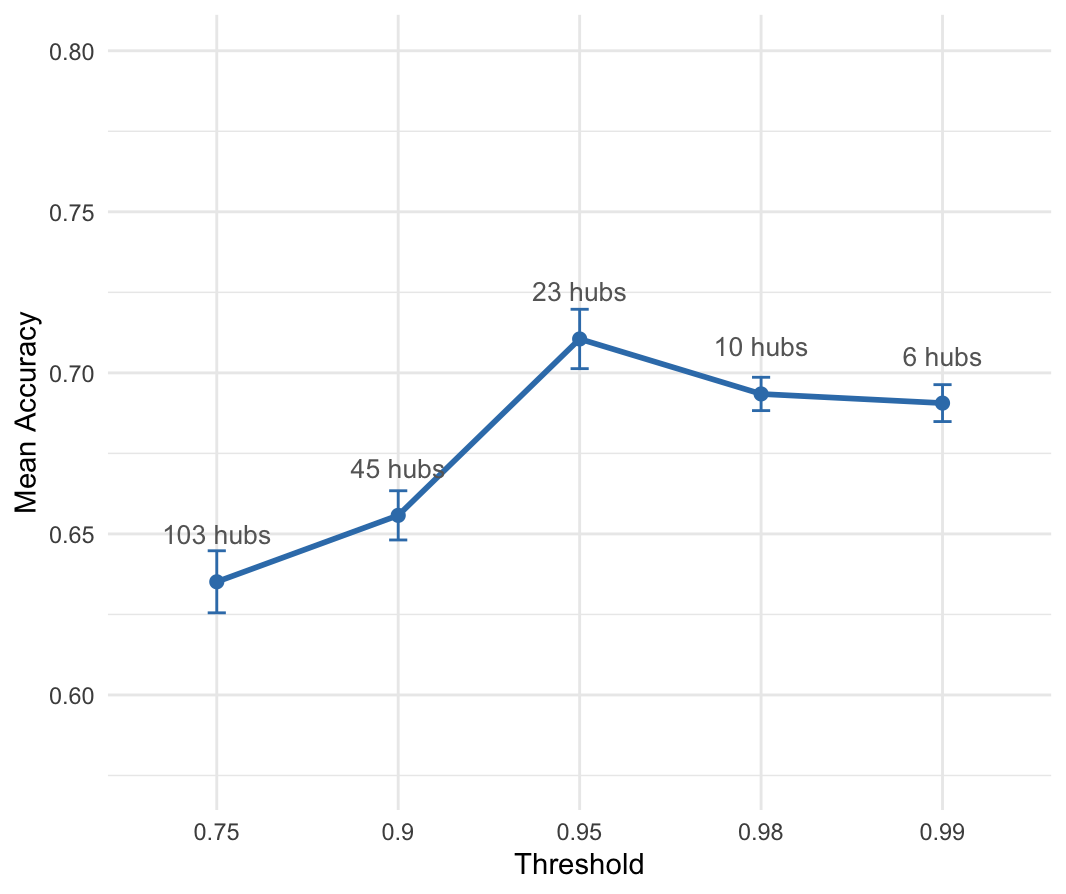


**Supplementary Figure 8. Leave-One-Out Cross Validation: plot of the mean accuracy (concordance with clinical response) with 95% CI for different percentile thresholds on the degree distribution of the differential co-expression network.**


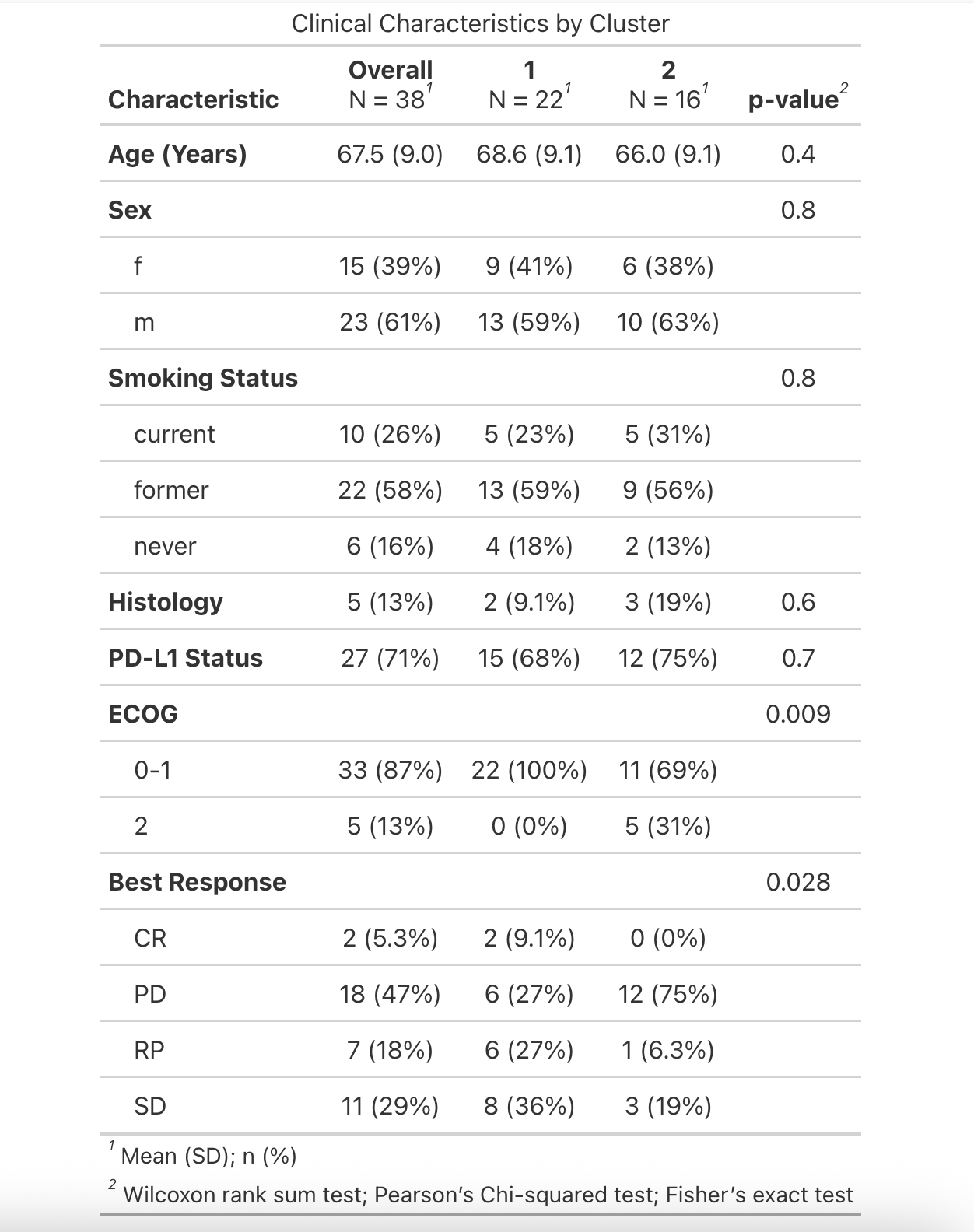


**Supplementary Table 7. Association between Network Clusters and baseline clinical characteristics.**


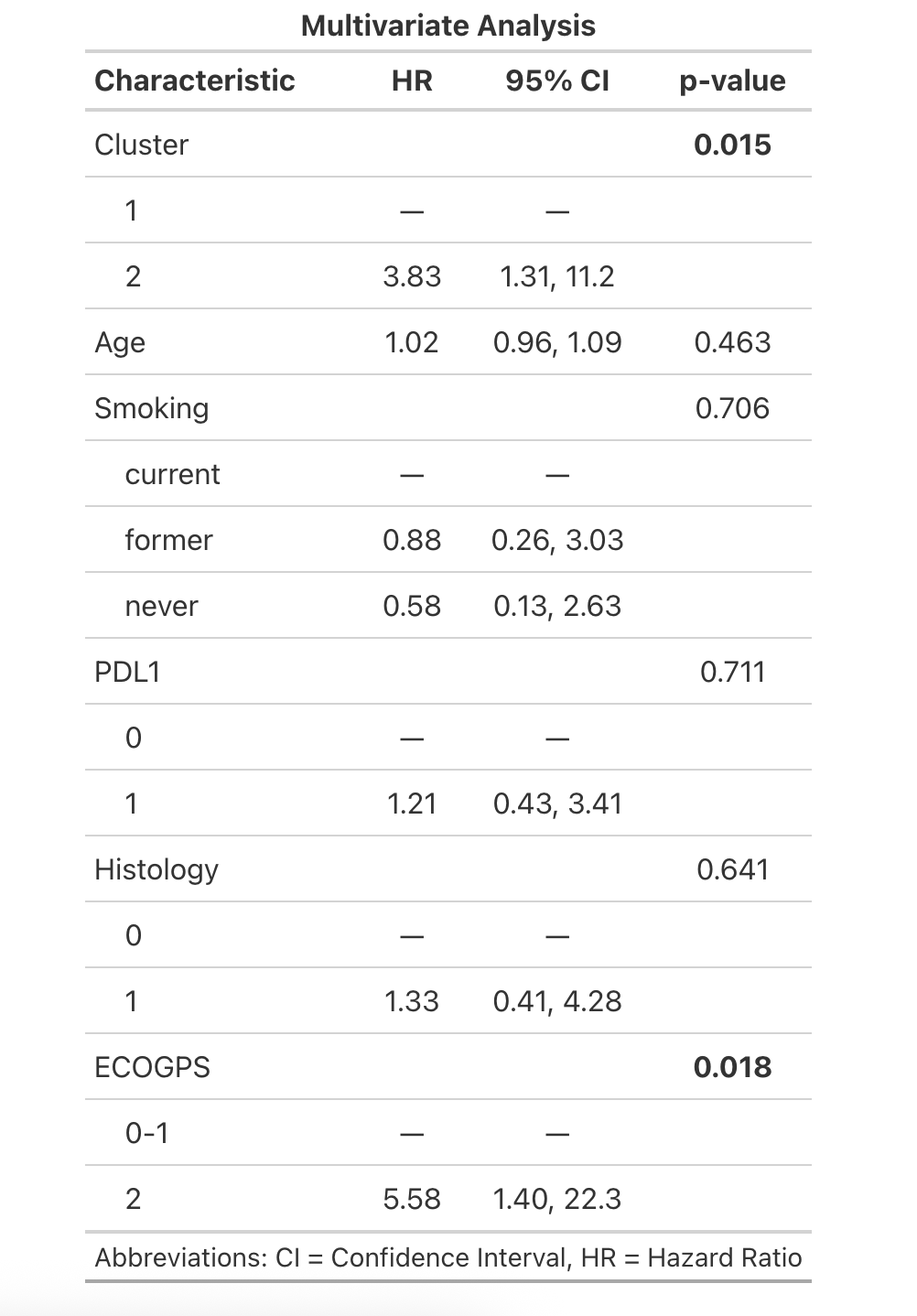


**Supplementary Table 8. Multivariate Cox proportional hazards regression analysis.**


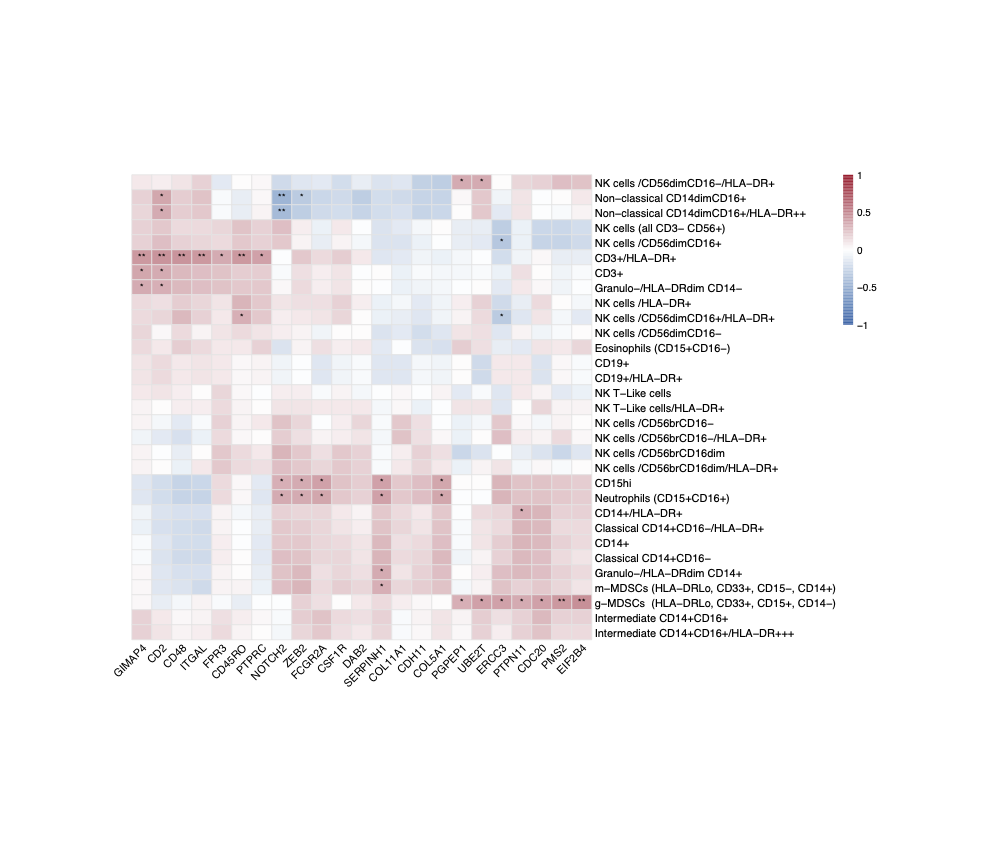


**Supplementary Figure 9. Correlation Heatmap showing Spearman Correlation between circulating immune profile (CIP) and DCN hub genes (GEP). Significance level: *** p < 0.001, ** p < 0.01, * p < 0.05.**

**Supplementary Part 2 - Materials and Methods**

## **Circulating immune profiling**

The methodology for circulating immune profiling (CIP) has been already extensively described in a previous publication [4]. Absolute cell counts of 36 immune subsets in peripheral blood were obtained using flow cytometry. Blood was collected at baseline and at day 63±3 days (first radiological evaluation) and stained with fluorescently labeled antibodies in Trucount Absolute Counting Tubes (Becton Dickinson). Samples were analyzed using a 10-color cytometer and data were analyzed with FlowJo software. Beads were gated out and peripheral blood mononuclear cells (PBMCs) were gated using side scatter (SSC) versus CD45 dot plots. Absolute cell count was calculated using the formula A=X/Y×N/V, where X=number of positive cells events, Y=number of bead events, N=number of beads in the test tube and V=test vol. Validation of flow cytometry cell counts data was obtained through correlation analysis with counts of lymphocytes, granulocytes and monocytes generated on the same samples by an automated hematology cell counter. Thirty-six distinct immune cell subsets were identified and counted using the gating strategy described in Lo Russo et al [4].

## **Transcriptomic profiling**

Total RNA was extracted from formalin-fixed, paraffin embedded (FFPE) tumor tissue at baseline, using the RNeasy FFPE Kit (Qiagen, Maryland, USA), according to manufacturer’s instructions. RNA 150 ng was used for gene expression analysis performed by means of the nCounter PanCancer Immune IO 360 Panel (NanoString). The 770-plex assay panel contains: 109 genes to cell surface markers capable of quantitating 24 different tumor infiltrating immune cell types and populations, 30 genes for commonly studied antigens, over 500 genes for measuring immune response with a special emphasis on checkpoint regulation/signaling and 40 PanCancer reference genes.

## **Data preprocessing**

All analyses were conducted using R software and specific visualizations were created using Cytoscape.

Out of the initial set of 36 immunological variables, a subset of 31 was chosen for the analysis. CIP data were available for 57 patients at baseline and only for 46 patients at first radiological evaluation, due to scanty samples or sudden progressive disease (PD). From the total of 57 patients, only those with data available at both baseline and post-treatment were selected, leaving only 45 patients, 23 responders - complete response (CR), partial response (PR) and stable disease (SD) - and 22 non-responders – PD.

Gene expression profiling (GEP) data were available only at baseline for 48 patients. Among these 48 patients, response information was available for 44 patients, comprising 25 responders and 19 non-responders. The NanoStringNorm package for R software was used to assess quality and process the raw counts; the geometric mean of the counts relative to each sample, the mean plus two SD and the total sum of counts options were used to correct the data for technical, background and batch effect issues, respectively. The expression counts of housekeeping genes and quantile normalization were used to account for inter sample variations with the panel. The analysis focused exclusively on 752 "endogenous" genes within the gene panel and genes with zero expression levels were excluded from the analysis, ensuring that the investigation concentrated on actively expressed genes. In this way, the analysis was conducted using 438 genes. Data were standardized.

The available data were categorized into four specific groups based on response and the timing of data collection: pre-therapy responders, post-therapy responders, pre-therapy non-responders and post-therapy non-responders.
